# A new knockin mouse carrying the E364X patient mutation for CDKL5 deficiency disorder: neurological, behavioral and molecular profiling

**DOI:** 10.1016/j.heliyon.2024.e40165

**Published:** 2024-11-06

**Authors:** C. Quadalti, M. Sannia, N.E. Humphreys, V.A. Baldassarro, A. Gurgone, M. Ascolani, L. Zanella, L. Giardino, C.T. Gross, S. Croci, I. Meloni, M. Giustetto, A. Renieri, L. Lorenzini, L. Calzà

**Affiliations:** a Department of Pharmacy and Biotechnology, University of Bologna, Bologna, Italy; b IRET Foundation, 40064 Ozzano Emilia (Bologna), Italy; c Epigenetics & Neurobiology Unit, European Molecular Biology Laboratory (EMBL), Rome, Italy; d Department of Veterinary Medical Sciences, University of Bologna, 40064 Bologna, Italy; e Department of Neuroscience “Rita Levi-Montalcini”, University of Turin, 10125 Turin, Italy; f Medical Genetics, University of Siena, 53100 Siena, Italy; g Medical Genetics Department, Siena University Hospital, 53100 Siena, Italy

**Keywords:** CDKL5, CDD, CRISPR/Cas9 knockin, GABA, Gabra1, Gabra5

## Abstract

CDKL5 deficiency disorder (CDD) is a rare neurodevelopmental syndrome caused by mutations in the X-linked CDKL5 gene. Hundreds of pathogenic variants have been described, associated with a significant phenotypic heterogeneity observed among patients. To date, different knockout mouse models have been generated. Here we present a new knockin CDKL5 mouse model carrying a humanized, well-characterized nonsense variant (c.1090G > T; p.E364X) described in the C-terminal domain of the CDKL5 protein in a female patient with a milder phenotype. Both male and female *Cdkl5*^E364X^ mice were analyzed. The novel *Cdkl5*^E364X^ mouse showed altered neurological and motor neuron maturation, hyperactivity, defective coordination and impaired memory and cognition. Gene expression analysis highlighted an unexpected reduction of *Cdkl5* expression in *Cdkl5*^E364X^ mice brain tissues, with a significant increase in overall neuron-specific gene expression and an area-dependent alteration of astrocyte- and oligodendrocyte-specific transcripts. Moreover, our results showed that the loss of CDKL5 protein had the most significant impact on the cerebellum and hippocampus, compared to other analyzed tissues. A targeted analysis to study synaptic plasticity in cerebellum and hippocampus showed reduced *Gabra1* and *Gabra5* expression levels in females, whereas *Gabra1* expression was increased in males, suggesting an opposite, sex-dependent regulation of the GABA receptor expression already described in humans. In conclusion, the novel Cdkl5^E364X^ mouse model is characterized by robust neurological and neurobehavioral alterations, associated with a molecular profile related to synaptic function indicative of a cerebellar GABAergic hypofunction, pointing to *Gabra1* and *Gabra5* as novel druggable target candidates for CDD.

## 1 Introduction

*Cyclin-dependent kinase-like 5* (*CDKL5*, OMIM:300203, 300672) deficiency disorder (CDD, OMIM #300203) is a rare encephalopathy that affects between 1:40 000 to 1:60 000 newborns, with females making up about 90 percent of diagnosed patients [1,2]. The International CDKL5 Disorder Database has been set up to gather data on all known CDD cases, and to promote the characterization of this little-known condition (www.telethonkids.org.au/projects/international-cdkl5-disorder-database/). CDD is a severe neuro developmental disorder characterized by early-onset epilepsy, severe cognitive impairment, jerky movements, central blindness, sleep disturbances and Rett-like stereotypies [2,3]. “Lumpers” - those who prefer broader categories despite some differences - have considered it a variant of Rett syndrome [4], while “splitters” - those who prefer narrower categories, emphasizing variations rather than common features - have recognized it as an independent disorder [5].

CDD is caused by mutations in the X-linked gene *CDKL5*, located on the Xp22.13 cytogenetic band and subject to random X inactivation in females [6]. It encodes a serine/threonine kinase which is widely distributed throughout the human body with the highest expression in the brain, predominantly in neuron nuclei and dendrites, peaking during early postnatal life, a period which corresponds to the typical onset of symptoms [1].

The CDKL5 kinase is characterized by a highly conserved N-terminal catalytic domain, which drives the fundamental role of this protein in brain development and function. Changes in the level of CDKL5 protein during development suggest an important role in the process of neuronal formation, maturation, morphogenesis and plasticity [2,7], and CDKL5 has been associated with neuronal migration, axonal outgrowth, dendritic morphogenesis and synapse formation [8]. Interestingly, post-developmental loss of CDKL5 has been linked to a disruption of behavior in several domains, as well as alterations in hippocampal circuits and dendritic spine morphology, highlighting the central role of this kinase not only during brain development, but also in brain maintenance and function. There is also growing evidence for the potential of at least a partial CDD reversal, opening a new therapeutic time window for potential treatment [[9], [10], [11], [12]].

Hundreds of pathogenic variants, including deletions, truncations, splice and missense mutations, have been described in the *CDKL5* gene to date [1,13], and significant phenotypic heterogeneity may be related to the type and location of the variant and the impact of epigenetic and environmental factors [1,13]. Studies show that patients with pathogenic variants within the N-terminal catalytic domain and frameshift mutations located at the end of the C-terminal region have more severe motor impairment, refractory epilepsy, or microcephaly [14]. No effective cure is currently available for this devastating neurodevelopmental disorder, making the development of molecular and genetic therapeutic options an issue of vital importance for patients and their families.

Several *Cdkl5* knockout (KO) and conditional KO mice [[15], [16], [17], [18]] have been generated and characterized to investigate the role of this kinase in the etiology of CDD. These models have been obtained through a large deletion in one of the exons coding for the N-terminal catalytic domain, mimicking the loss of function of the protein. Researchers have recently described *Ckdl5* knockin (KI) and conditional KI mouse models carrying a well-characterized patient variant (p.R59X) [19,20], a pathogenic nonsense mutation localized in the N-terminal catalytic domain of CDKL5 which results in the absence of the protein in KI mouse models [19,20].

While the overwhelming majority of available studies on mouse model characterization to date have been based on hemizygous male subjects to avoid any potential confounding effects linked to X inactivation in heterozygous female mice [15,[17], [18], [19],^21^], in humans CDD is more common in females than in males, with only 50 cases of CDD described in males to date [22] presenting a more severe phenotype than female patients.

In this paper, we generated and characterized a new CDD mouse model carrying a humanized, well-characterized nonsense variant (c.1090G > T; p.E364X) found in the C-terminal domain of the CDKL5 protein of patients that presented with a milder phenotype and suitable for gene editing correction [23]. The goal of the neurological, behavioral and molecular characterization of this new *Cdkl5*^E364X^ mouse model is to unveil robust functional and/or behavioral alterations and the related molecular profiles, in order to identify potential endpoints for gene therapy monitoring. To reach this goal, we characterized all the animals born in the generated colonies, both male and female mice of all genotypes, namely hemizygous (HEM) and wild type (WT) males, heterozygous (HET), homozygous (HOM) and wild type females, for the neurological maturation (up to 21 postnatal days) and the standard neurobehavioral testing in adult life (80 and 120 days). Then, as no differences were observed between heterozygous and homozygous, in order to reduce the possible bias associated with the high variability usually observed among heterozygous and thus better identify a potential therapeutic endpoint, the most demanding behavioral test (Morris water maze, 200 days) and the molecular and immunofluorescent characterization were performed in all male genotypes and in WT and homozygous females only.

## 2 Materials and methods

### 2.1 Animals

To generate a mouse model that could potentially be suitable also for gene therapy testing, a *Cdkl5*^*E364X*^ KI mouse model was generated that harbors a pathogenic variant identified in a CDD patient. The model is produced by the insertion of a humanized region of 46 bp surrounding the pathogenic variant of interest (c.1090G > T, g.18622134G > T). The chosen nonsense mutation causes the change of one amino acid in the protein sequence, p.E364X, also known as p.Glu 364∗ (http://mecp2.chw.edu.au/cdkl5/cdkl5_variant_list_copy.php).

The mutant Cdkl5 allele was created by CRISPR/Cas9-editing technology using C57BL/6J (Charles River) zygotes as previously described [24]. Briefly, for *Cdkl5*^*E364X*^ (C57BL/6JCrl-Cdkl5^em1(E364X)^Emr) a CRISPR crRNA oligo (CGTTTGCTTTCGAAGGAGTT) was annealed with tracrRNA and combined with a homology flanked ssODN donor coding for STOP at amino acid position 364, substituting the wild type glutamic acid. The target location was exon 12 of transcript Cdkl5-201, (ENSEMBL v109); genomic coordinate ChrX. 159617718 (GRCm39/mm39). Additionally, the ssODN donor recoded the adjacent genomic region (46 bps, ChrX. 159617720–159617766) altering the sequence to match human *CDKL5* (ChrX. 18604014-18603968, GRCh38.p14). See Supplementary Fig. S1A for the *in silico* design.

The annealed sgRNA was complexed with Cas9 protein and combined with the ssDNA donor (Cas9 protein 50 ng/μL, sgRNA 20 ng/μL, ssDNA 20 ng/μL). All single-stranded DNA and RNA oligos were synthesized by IDT (Integrated DNA Technologies, Inc., Iowa, USA). Cas9 protein, sgRNA and ssDNA donor template were co-microinjected into zygote pronuclei using standard protocols [25] and after overnight culture, 2-cell embryos were surgically implanted into the oviduct of day 0.5 *post-coitum* pseudopregnant CD1 mice. Founder mice were screened for the presence of the mutation by PCR, which was then confirmed via Sanger sequencing and aligned with the *in silico* design.

Transgenic mouse production was performed by the Gene Editing & Embryology Facility at EMBL, Rome. The mutant strain was maintained on a C57BL/6JCrl genetic background. PCR genotyping of the mice tails was performed at weaning, at postnatal day (PND) 21. Mice were produced at EMBL and transported to Bologna for breeding and phenotypic testing.

Mating of *Cdkl5* heterozygous (X/+) females with *Cdkl5* hemizygous (+/Y) and *Cdkl5* wild-type (X/Y) males allowed the generation of wild-type (WT X/X), heterozygous (HET X/+) and homozygous (HOM +/+) females and wild-type (WT X/Y) and hemizygous (HEM +/Y) males.

### 2.2 Genotyping

Genomic DNA was extracted using the PCRBIO Rapid Extract PCR Kit (Resnova, Rome, Italy), following the manufacturer's instructions. Briefly, tail tissue of 1–2 mm was added in the extraction buffer (Buffer A, 20 μl; Buffer B, 10 μl; water, 70 μl) and incubated at 75 °C for 5 min and then at 95 °C for 10 min. The digested sample was transferred to a new tube and 900 μl of water were added. Digested samples were then centrifuged at 8000×*g* for 1 min. The supernatant was transferred to a new tube and used for PCR, while the pellet containing tissue debris was discarded.

The PCR was performed in a 25 μl reaction volume per sample, using the PCRBIO HS Taq Mix Red (12.5 μl), the specific primers (FW: CCTTCCCATTCTCCAGCTCT; REV: AGTGCCTGTTCTGCTGAGAT; 1 μl–400 nM each), water (8.5 μl) and 2 μl of the digested sample supernatant. The reaction was performed using the following thermal profile: 95 °C for 1 min; 35 cycles of 95 °C for 15 s, 60 °C for 15 s, and 72 °C for 30 s. Duplicate reactions were performed for each animal, and for each session a no-template control (NTC, where no DNA was added) was included to check for contaminations.

The PCR amplification products (10 μl each) were used for the enzymatic digestion with *Mlu*CI enzyme (10 U; New England BioLabs, Ipswich, MA, USA) at 37 °C for 1 h. The correct insertion of the mutation of interest generates a unique cut site for *Mlu*CI enzyme, otherwise absent, allowing the discrimination of the *Cdkl5*^*E364X*^ from the *Cdkl5*^*WT*^ genotypes.

Enzymatic digestions were evaluated by electrophoresis run on a 1 % agarose gel or gel separation analysis performed with QIAxcel instrument and DNA High Resolution Kit (Qiagen, Hilden, Germany). For each sample, a duplicate of both the digested and the non-digested PCR product were loaded. Moreover, for each reaction, positive controls for the different genotypes were processed in the same way, to check that all the reactions in every step worked properly.

The genotypes were easily identified by the presence of specific bands according to the sex of the animal. Both female WT (X/X) and male WT (X/Y) were identified by a single band corresponding to the WT non-digested gene (532 bp); male HEM (+/Y) showed two bands corresponding to the specific enzymatic digestion of the mutated gene (250 bp and 282 bp); female HET (X/+) displayed both the WT band (532 bp) and the two digestion products (250 bp and 282 bp), while female HOM (+/+) showed only the digested bands (250 bp and 282 bp) (Supplementary Fig. S1C).

### 2.3 Neurological maturation and neurobehavioral tests

Neurological development was analyzed in all mice pups from P7 to P21 between 10 and 12 a.m., every second day. Pups were tested for righting reflex, negative geotaxis, grip strength, grasping reflex, cliff aversion, eyes open, gait and clasping, sensory reflex and auditory startle [26]. All measurements were acquired in triplicate and the mean of the three repetitions was used as individual value for statistical analysis.

The righting reflex is an innate reflex indicating the capability of a newborn to move from the supine to the prone position (“flip” movement). Negative geotaxis is an automatic response of pups placed upside down in the middle of a 30 cm slope (45°), measured by recording the latency to turn 180° to an upward direction, expressed in seconds.

Sensory reflex test is evaluated by touching the ear and the eyelid of the pups with a cotton swab, and the first day of respective reflex appearance is recorded. Auditory startle test consists in recording the first day of the startle response to a clapping sound.

The first day of lifting and placing the paws on the table is recorded. Limb grasping reflex is under the control of nonprimary motor areas to spinal interneurons via by a spinal reflex mechanism. It is assessed by touching the forelimbs with a thin rod. The first day of grasping onto the rod is recorded.

Irregularities in gait development are measured by placing the animals at the center of a white plexiglass circle (Ø = 13 cm) and measuring the time needed to exit the circle with both front paws. The test is considered negative when exceeding 30 s.

The cliff aversion test evaluates the labyrinthine postural reflex, as well as strength and coordination. Pups are placed on the border of a table having nose and forelegs over the edge, and result is expressed as cliff avoidance by backing away, turning sideways, or moving away from the edge [26,27]. The mouse is considered unable when exceeding 30 s.

Eyes open test is the on-off observation of both left (L) and right (R) eyes opening in newborns (0 = closed, 1 = opened).

For some tests it is only possible to evaluate the day of appearance (eye open, sensory and auditory reflex and grasping reflex), while for righting reflex, negative geotaxis and gait, it is possible to quantify the performance (from 0 to 3) at each time-point. The scores are calculated according to the latency for the execution of the test, following the criteria indicated in Table 1. Cliff aversion is evaluated both as day of appearance and as scoring performance. The sum of all scores at each time points is presented as “neuroscore”. Higher scores indicate better development of neurological reflexes.

**Table 1:** Neuroscore calculation criteria.

Score	Range [seconds]
0	>30
1	21 to 30
2	11 to 20
3	0 to 10

Clasping is a functional motor test to evaluate limb positioning in a mouse suspended by its tail. Hind limb closure (hind limbs pulled back toward the abdomen) is an early indicator of neurodegenerative disease progression, and it quantifies impairment in corticospinal function by observing limb extension [28,29]. The reflex is visible after a few seconds and evaluated from 0 to 4 [30], as summarized in Table 2 (see Supplementary Fig. S2 for a representative image of score 0 and score 4 clasping behaviour).

**Table 2:** Clasping score criteria.

Score	Sign description
0	No clasping. Normal limb extension
1	One hind limb exhibits incomplete extension
2	Both hind limbs exhibit incomplete extension
3	Both hind limbs exhibit incomplete extension accompanied by curled toes
4	All four limbs, fore and hind together, are closed inward with curled toes

In 80 and 120-day-old mice, locomotion (Spontaneous Locomotion, Rotarod and Gait Analysis) and spatial working memory (Y-maze) have been evaluated. Morris Water Maze test has been performed in 200-day-old mice [[31], [32], [33]].

Spontaneous locomotion is assessed in a test arena consisting of a 46 x 46 × 41 cm plastic chamber, with a dark grey floor, virtually divided into 16 fields by AnyMaze Video tracking software (AnyMaze, Stoelting-Wood Dale, IL). Mice are individually placed in the center of the chamber, always facing the same direction. Distance traveled and speed over 10 min are measured using AnyMaze Video tracking software.

The Rotarod test (LE 8500 RotaRod: 2 Biological Instruments, Varese, Italy) consists of two days of testing. On day 1, a training session consisting in three trials (maximum 5 min each) at constant speed (5 rpm, 10 rpm and 15 rpm) was administers. On day 2 (24 h later), mice were placed on the progressively accelerating drum and the latency to fall was measured by the SEDACOM32 program version 1.0 (PanLab, Barcelona, Spain) [31]. The acceleration rate of the rotarod is from 16 to 40 rpm over a 5 min period.

Gait was analyzed by CatWalk system (Noldus Information Technology, Wageningen, The Netherlands), composed by a glass walkway and a camera recording the mouse gait from the floor. The glass walkway is illuminated with beams of light, thereby allowing the animals' paws to reflect light as they touch the glass floor. Each mouse performed three complete runs, and the average values are used for the statistical analysis. If an animal fails to complete a run within 5 s, walks backwards or rear during the run, the process is aborted and repeated. Each paw is labeled on the recorded video in order to calculate paw-related parameters. The gait-related parameters measured using the CatWalk apparatus and software (Catwalk XT. Version 10.5, Noldus Information Technology, Wageningen, Netherlands) are the following: the maximum contact area (paw area that comes into contact with the glass plate), the stride length (distance between successive placements of the same paw) [31], the base of support (BOS, average width between either the front paws or the hind paws), the Duty cycle (percentage of step cycle), the regulatory index (number of normal step sequence patterns relative to the total number of paw placements calculated as ((normal step sequence patterns ∗ 4)/total number of paw placements) ∗ 100))) and the cadence (steps per second).

The Y-maze consists of a Y-shaped maze (arms A-B-C) where mice are placed at the beginning of arm A and left free to explore the three arms of the maze for 8 min. The total number of entries in the maze arms is recorded and the percentage of correct alternations is calculated as follow: % of alternations = (total number of alternations/number of arms entries) × 100 [34].

The spatial memory was evaluated using Morris Water Maze (MWM) test, using a 120 cm-diameter pool with 45 cm high walls, virtually divided in four equal quadrants by the AnyMaze software (Any-Maze Video Tracking Software v. 6.3 – Stoelting Co., Wood Dale, U.S.A.). The water of the pool is made opaque by the addition of rice starch. The water level is 1 cm above a transparent plexiglass platform (10 cm diameter). On the first day, mice were gently positioned on the platform for 30 s, to allow them to explore it and observe the different cues around the perimeter of the pool and on the pool walls (proximal and distant cues). Mice are then trained to reach the hidden platform. The first phase of this test consists in a pre-training of two consecutive days, with three trials per day. In each trial, the mouse is immersed in the pool in a different starting point and the test stopped when mice reach the visible platform marked by two flags. The second phase consists in the acquisition, consisting in three trials per day for six consecutive days using submerged hidden platform, without flags but with proximal and distant cues. In each trial, the mouse is immersed in the pool in different starting point and the test stops when mice reach the platform. Mice that failed to reach the platform within 60 s were gently accompanied to the platform by the operator to reinforce habituation, in both pre-training and acquisition phases. The inter-trial interval for both phases is 30 min.

To assess long-term memory, a probe trial 24 h after the last acquisition trial is performed without platform, and a 60-s free swim is allowed. Number of entries, time spent and latency (in seconds) to first entry in platform zone are recorded [32].

### 2.4 Gene expression analysis

For gene expression analysis, 6-months-old males, WT (X/Y) and HEM (+/Y), and females, WT (X/X) and HOM (+/+), were included (n = 5 per group). The total RNA was extracted from the homogenized tissues using the RNeasy Plus Universal Mini Kit (Qiagen, Hilden, Germany) following the manufacturer's instructions and quantified by Nanodrop 2000 spectrophotometer. Then, according to the aim of the experiment, different protocols were used to produce the cDNA and to perform the qPCRs.

First, we analyzed the expression of the murine *Cdkl5* gene, designing the primer pair before the humanized insertion containing the pathogenic variant of interest, allowing the quantification of the *Cdkl5* mRNA, regardless of the presence of the insert. In particular, the humanized sequence was inserted in the protein coding sequence, modifying the 12th exon, while the primers pair was placed between the 6th and 9th exons (Supplementary Fig. S1B). For *Cdkl5* gene expression level, four different areas of the brain were employed (cortex, CTX; cerebellum, CB; hippocampus, HIP; spinal cord, SC) and two reference tissues were selected to account for high-expressing (testis) or low-expressing (liver) tissues, as described in the human protein atlas (https://www.proteinatlas.org/ENSG00000008086-CDKL5/tissue). After total RNA extraction, cDNA was retrotranscribed using the iScript™ gDNA Clear cDNA Synthesis Kit (Bio-Rad Laboratories, Segrate, MI, Italy), starting from 1 μg of RNA, following the manufacturer's instructions. A no-reverse transcription sample was added, using the iScript Supermix No-RT Control instead of the reverse transcription enzyme, and processed for gene expression analysis in parallel with the other samples to check for genomic DNA contaminations. The qPCR reaction was performed using the CFX96 machine (Bio-Rad) and the SsoAdvanced™Universal SYBR®Green Supermix (Bio-Rad), starting from 10 ng of cDNA. Relative quantification of mRNA was obtained through the comparative cycle threshold (Ct) method. Ct values were collected for each sample, normalized first on the housekeeping gene actin beta (Actb), and represented as a normalization on the male WT (X/Y) group, calculated as 2^(-ΔΔCt).

The same protocol was used to quantify the expression of the lineage-specific genes, enolase 2 (*Eno2*) for neurons, glial fibrillary acidic protein (*Gfap*) for astrocytes, platelet derived growth factor receptor alpha (*Pdgfra*) for oligodendrocyte precursor cells (OPCs), and myelin basic protein (*Mbp*) for mature myelinating oligodendrocytes. For the central nervous system (CNS) analysis, Glyceraldehyde-3-phosphate dehydrogenase (*Gapdh*) was used as housekeeping gene (the specific primer sequences are included in Table 3).

**Table 3:** Gene specific primer sequences used for RT-qPCR analysis.

Gene	Forward primer *s*equence (5′-3′)	Reverse primer sequence (5′-3′)
Cdkl5	CTGGTGCCACAAGAACGACA	TTCCATAGGGGGCTCCAAGTA
Eno2	AATCAGATCGGCTCGGTCAC	TCCTCAATTCTCATGAGCTGGT
Gfap	AGTGGTATCGGTCCAAGTTTGC	TGGCGGCGATAGTCATTAGC
Pdgfra	CGGAACCTCAGAGAGAATCGG	TCCCCATAGCTCCTGAGACC
Mbp	GCCTGTCCCTCAGCAGATTT	GTCGTAGGCCCCCTTGAATC
Gad65	GCCCGCTATAAGATGTCTC	AATCACACTGTCTGTTCC
Gad67	GGCATCTTCCACTCCTTC	GACGACTCTTCTCTTCCAG
Gat1	AAGGGTGTTGGTTGGACTGG	AGCCACACCTCAGAATCAGAC
Vgat	GAGGAAGACCCCAGGTAGC	GGGTCACGACAAACCCAAGA
Gabra1	CACCATGAGGTTGACCGTGA	TACAACCACTGAACGGGCTG
Gabra5	CCCTCCTTGTCTTCTGTATTTCC	TGATGTTGTCATTGGTCTCGTCT
Actb	CTGTCGAGTCGCGTCCA	TCATCCATGGCGAACTGGTG
Gapdh	GGCAAGTTCAATGGCACAGTCAAG	CATACTCAGCACCAGCATCAC

The same RNAs were used to analyze the expression of genes involved in synaptic plasticity in hippocampus and cerebellum, through the PCR array technology. For cDNA synthesis, the RT^2^ first strand kit (Qiagen) was used, starting from 1 μg of pooled RNAs from each experimental group. Eighty-four genes involved in the synaptic plasticity process were analyzed using the commercial RT^2^ PCR array PAMM-126Z (Qiagen), which also included five common housekeeping genes and quality controls for genomic DNA contamination, reverse transcription reaction and inter-array reproducibility. Following the manufacturer's protocol, the real-time PCR was performed using the RT2 SYBR Green qPCR Mastermix (Qiagen) on the CFX96 machine (Bio-Rad). For both hippocampus and cerebellum analysis the quality controls were passed by the whole sample sets.

Data were analyzed using the GeneGlobe online platform (https://geneglobe.qiagen.com/us/analyze) (Qiagen). According to the Ct data, the software selected specific housekeeping genes among the five genes included in the array for the analysis in the hippocampus (Beta-2-microglobulin, *B2m*; Glucuronidase beta, *Gusb*; Heat shock protein 90 alpha family class B member 1; *Hsp90ab1*) and in the cerebellum (*Gapdh*; *Gusb*; *Hsp90ab1*). The full list of the analyzed genes is included in the supplementary material (Supplementary Table S1).

Gene expression variation was analyzed as ΔCt to produce the clustergrams, showing the distribution of the expression of each gene across the groups and their clusterization according to the whole gene set. Relative gene expression was also analyzed, normalizing the ΔCt on a reference group to produce the scatter plots indicating the expression variation between groups, using a cut-off of 2-fold-change.

To validate the PCR array data produced on pooled samples, we performed additional qPCR analysis using cDNAs from single animals, as described for the lineage-specific genes, on genes involved in the gamma-aminobutyric acid (GABA) and glutamate pathways: glutamate decarboxylase isoform 65 kDa, *Gad65*; glutamate decarboxylase isoform 67 kDa, *Gad67*; GABA transporter 1, *Gat1;* vesicular GABA transporter, *Vgat;* GABA type A receptor subunit alpha 1, *Gabra1*; GABA type A receptor subunit alpha 5, *Gabra5*. The list of the specific primers is included in Table 3. Results were analyzed as described above, using *Gapdh* as housekeeping gene, while normalizing as control group the WT expression of the respective sex.

### 2.5 Histological and immunofluorescence procedures

For morphological analysis, mice were anesthetized with tiletamine/zolazepam (40 mg/kg) and xilazine (4–5 mg/kg) and transcardially perfused with 10 ml of 0.1M PBS followed by 80 ml of ice-cold 4 % paraformaldehyde in 0.1M PB. Brains were quickly dissected, and kept in fixative solution overnight at 4 °C, cryoprotected by immersion in raising sucrose-PB 0.1M solutions (10, 20 and 30 %), cut into 30 μm coronal sections with a cryostat and stored at −20 °C in a cryoprotectant solution containing 30 % ethylene glycol and 25 % glycerol until use [35].

For gross histological observations, brains were coronally sectioned using a cryostat (Leica CM1950, 14 μm thick section), stained using toluidine blue, and observed using a Nikon FXA microscope equipped with a Nikon Digital camera DXM1200F.

For immunofluorescence, free-floating sections were washed twice with 0.1M PBS before antigen-retrieval was performed by incubation with mild agitation in 0.01M sodium citrate (pH 6.0) at 75 °C for 45 min [36]. Sections were then washed twice with 0.1M PBS and subsequently processed by immersion in 0.1M PBS solution containing 3 % normal donkey serum (NDS) and 0.5 % Triton X for 1h followed by O/N incubation at 4 °C with CDKL5 primary antibody (sheep anti-human CDKL5; S957D polyclonal antibody, University of Dundee). The following day, sections were rinsed with 0.1M PBS, incubated for 1h at RT with a fluorescent secondary antibody (anti-sheep 1:1000; Jackson ImmunoResearch, West Grove, PA, USA) and cell nuclei were stained with DAPI. The sections were then washed three times with PBS, mounted on gelatin-coated glass slides and cover slipped with Dako fluorescence mounting medium (Dako Italia, Italy).

### 2.6 Confocal microscopy images acquisition

Immunofluorescence images of the primary somatosensory cortex, from the pial surface to the corpus callosum, and area CA1 of the hippocampus were acquired with a laser scanning confocal microscope (LSM 900; Zeiss, DE; and Axio Imager.Z2; Zeiss, DE, Germany) using a 40 × objective (1.3 numerical aperture) and the pinhole set at 1 Airy unit [37]. Images of CDKL5 immunofluorescence were acquired by optimizing confocal microscope settings (i.e.: laser intensity, detector gain, and exposure time) on brain sections deriving from WT animals and then kept constant throughout each imaging session that always included sections from both WT (X/X) and HOM (+/+) female mice.

### 2.7 Brain lysates preparation

Mice were decapitated and brains rapidly collected on ice and frozen in liquid nitrogen. The cerebral cortices, the hippocampi and the cerebella were dissected and homogenized in lysis buffer (50 mM HEPES pH 7.0, 250 mM NaCl, 0.5 % NP-40, 5 mM EDTA, 1 mM DTT) added with 1 % protease and phosphatase inhibitors cocktail (0.5 mM Na3VO4, 0.5 mM PMSF and 50 mM NaF, Sigma). Homogenates were centrifuged for 45 min at 4 °C at 13000 rpm and supernatants collected and stored at −80 C. The protein content was determined using a Nanodrop (Thermofisher, Massachusetts, United States) analyzer.

### 2.8 Western blotting

Lysates were boiled in SDS sample buffer, separated by SDS–PAGE and the proteins were then blotted to PVDF membrane following a standard protocol [35]. Next, PVDF membranes were blocked in BSA 5 % for 1h and incubated with the primary antibodies (rabbit anti-Cdkl5, 1:250, Sigma-Aldrich cat. HPA002847, anti-mouse actin 1:1000 Immunological Sciences) O/N at 4 °C. After washes with TBS 0.1 % Tween 20, the membranes were incubated with the appropriate secondary antibodies (anti-rabbit, 1:5000; Sigma, Italy) for 1h at RT. The chemiluminescent signal was visualized using (Westar Antares, Cyanagen, Italy) and acquired with Bio-Rad ChemiDocTM Imagers (Bio-Rad; Italy).

### 2.9 Statistical analysis

The statistical analyses and graphs were performed using GraphPad Prism v10.2.2 (GraphPad Software, San Diego, CA, USA). All data are expressed as mean ± SEM. Data have been analyzed for normality using D'Agostino-Pearson test and outliers were identified and excluded (ROUT method, Q = 1 %). For normally distributed data, Student's t-test was used to compare the means between two groups, while one-way and two-way ANOVA (or mixed-effect model with Geisser-Greenhouse correction in case of missing data) and *post-hoc* tests were used to compare the means between three or more groups. For non-normal data, Mann-Whitney test was used to compare the means between two groups, while Kruskal-Wallis and *post-hoc* tests were used to compare the means between three or more groups.

The analysis performed in each experiment are described in the main text (Results section), where p values related to the overall test are reported, and in the respective figure legend, where asterisks related to the overall test (in case of comparisons between two groups) or to the corrected multiple comparisons (in case of three or more groups) are shown. Results were considered significant when the probability of their occurrence due to chance alone was less than 5 % (p < 0.05).

## 3 Results

### 3.1 Generation of a Cdkl5^E364X^ knockin mouse model

To assess the consequences of patient-specific CDKL5 variants in the pathophysiology of CDD and to obtain a model that could potentially be suitable for gene therapy testing, we generated a *Cdkl5*^*E364X*^ KI mouse model humanized for the presence of a nonsense mutation described in a patient [23] (Supplementary Fig. S1A). This pathogenic variant results in a premature termination codon in the early C-terminus tail of the CDKL5 kinase. Genotyping was performed as described in the Materials and Methods section, and tables detailing the generation of the murine colonies of interest can be found in Supplementary Table S2.

**Fig. 1:**
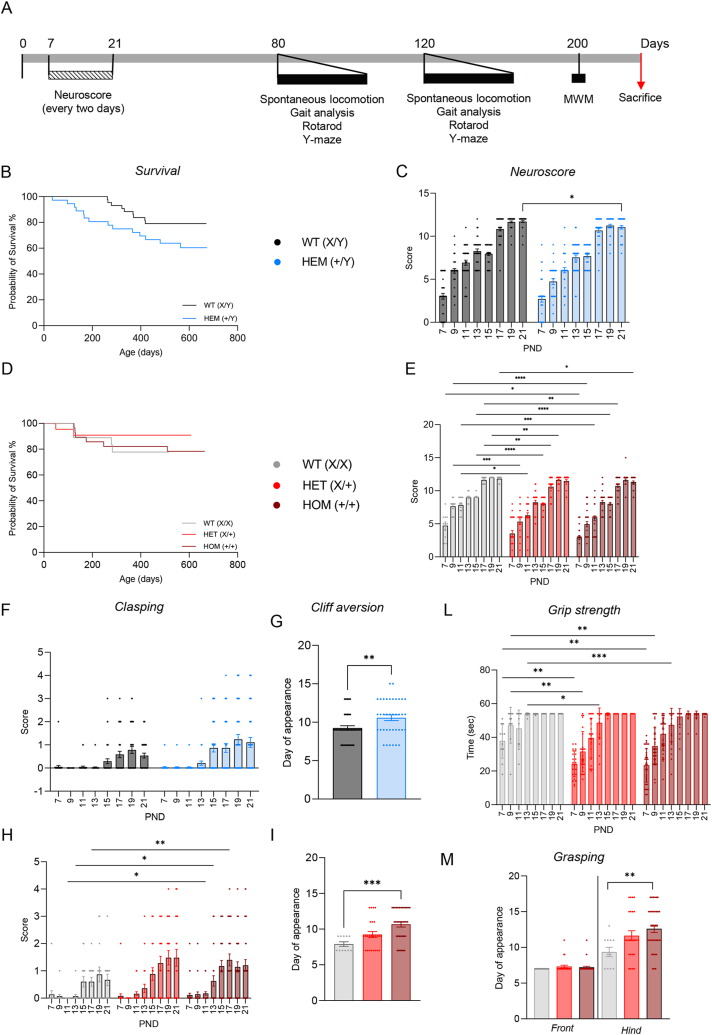
Colony survival and neurological maturation (A) Experimental design of neurological and behavioral monitoring of mice colonies. (B, D) Survival curve in male (B) and female (D) colonies. Curve comparison (Mantel-Cox and Gehan-Breslow-Wilcoxon tests) showed no statistical significance. (C, E) Neurological maturation. Neuroscores in WT males (C, n = 41) and females (E, n = 11) reach their maximum values faster than Cdkl5^E364X^ littermates (males HEM (+/Y), n = 38; females HET (X/+), n = 25; females HOM (+/+), n = 36); two-way ANOVA, factors: genotype and time (for statistical analyses data, see Result section); asterisks represent the multiple comparison analysis for the factor “genotype” with *post hoc* Sidak's test (males; day 21, WT (X/Y) vs HEM (+/Y), p = 0.0383) or Dunnett's test (females; day 7, WT (X/X) vs HOM (+/+), p = 0.0202; day 9, WT (X/X) vs HET (X/+), p = 0.0004, WT (X/X) vs HOM (+/+), p < 0.0001; day 11, WT (X/X) vs HET (X/+), p = 0.0124, WT (X/X) vs HOM (+/+), p = 0.0009; day 15, WT (X/X) vs HET (X/+), p < 0.0001, WT (X/X) vs HOM (+/+), p < 0.0001; day 17, WT (X/X) vs HET (X/+), p = 0.0037, WT (X/X) vs HOM (+/+), p = 0.0023; day 19, WT (X/X) vs HET (X/+), p = 0.0073; day 21, WT (X/X) vs HOM (+/+), p = 0.0380). (F, H) Hindlimb clasping is shown in both males (F) and females (H). The clasping score was significantly higher in male HEM (+/Y) mice (n = 38) than male WT (X/Y) mice (n = 41) (mixed-effect with Gaussier-Greenhouse correction, *post hoc* Sidak's test which showed no statistical significance among groups; for statistical analysis values, see Results section). Both Cdkl5^E364X^ female genotypes (HET (X/+), n = 25; HOM (+/+), n = 35) show a similar altered trend compared to their WT counterparts (n = 15), reaching statistical significance at PND 11 (WT (X/X) vs HOM (+/+), p = 0.0214), 13 (WT (X/X) vs HOM (+/+), p = 0.0134) and 17 (WT (X/X) vs HOM (+/+), p = 0.0092) (asterisks represents multiple comparisons significance with *post hoc* Dunnett's test; for statistical analysis values, see Results section). (G, I) Cliff aversion reflex in males (G) and females (I), evaluated on day of appearance. In both sexes, this reflex appears later in Cdkl5^E364X^ than in WT. Males HEM (+/Y) (n = 38) vs WT (X/Y) (n = 41), females HOM (+/+) (n = 36) and HET (X/+) (n = 25) vs WT (X/X) (n = 11). For statistical analyses values, see Result section. Asterisks represent the result of *t*-test in males and of Kruskal-Wallis multiple comparisons with *post hoc* Dunn's test in females (WT (X/X) vs HOM (+/+), p = 0.0008). (L) Grip strength test in females, evaluated in seconds, from PND 7 to PND 21. Females HOM (+/+) (n = 36) and HET (X/+) (n = 25) vs WT (X/X) (n = 11). For statistical analyses values, see Result section. Asterisks represent the result of mixed-effect model with *post hoc* Dunnett's test (PND 7, WT (X/X) vs HET (X/+), p = 0.0023, WT (X/X) vs HOM (+/+), p = 0.015; PND 9, WT (X/X) vs HET (X/+), p = 0.0014, WT (X/X) vs HOM (+/+), p = 0.0067; PND 13, WT (X/X) vs HET (X/+), p = 0.0139, WT (X/X) vs HOM (+/+), p = 0.0010). (M) Grasping reflex in females evaluated as day of appearance. Females HOM (+/+) (n = 36) and HET (X/+) (n = 25) vs WT (X/X) (n = 11). For statistical analyses values, see Result section. Asterisks represent the result of one-way ANOVA with *post hoc* Dunnett's test (hind paws, WT (X/X) vs HOM (+/+), p = 0.0058). ∗p < 0.05; ∗∗p < 0.005; ∗∗∗p < 0.001; ∗∗∗∗p < 0.0001. All data are expressed as mean ± SEM.

Breeding, nursing, and weaning were comparable among genotypes, as was body weight from birth to the end of the experimental period. While both male and female *Cdkl5*^*E364X*^ mice showed spontaneous mortality over the experimental time frame and a trend toward lower survival rates over the long term compared to the same-sex WT mice, the difference was not statistically significant (Fig. 1B–D).

### 3.2 Neurological and behavioral tests

The schedule of neurological and behavioral testing is shown in Fig. 1A. Neurological maturation was tested by reflex analysis between 7 and 21 postnatal days (PND), while locomotion, sensorimotor integrations and learning and memory tests were performed in young adult (80-day-old) and adult (120-day-old) mice.

### 3.3 Neurological maturation

All mice pups (both females and males, Cdkl5^E364X^ and WT) were evaluated for neurological maturation using a panel of tests comprising righting reflex, negative geotaxis, gait, grip strength, grasping reflex, eyes open, sensory reflex, auditory startle, cliff aversion and clasping. The tests explored sensorimotor and vestibular function, and the maturation of connections between the supplementary motor area, basal ganglia and the reticular formation, as well as possible muscle weakness [26], and significant results are shown in Fig. 1C (males) and 1E (females). The neuroscore (comprising righting reflex, negative geotaxis, gait and cliff aversion score) shows that WT animals of both sexes achieved their maximum scores faster than their Cdkl5^E364X^ littermates, a difference particularly significant in females (two-way ANOVA, males HEM (+/Y) vs WT (X/Y), factor “genotype”: p = 0.0020, F (1, 77) = 10.20; factor “time”: p < 0.0001, F (5.154, 396.9) = 355.0, interaction not significant; *post hoc* Sidak's test; females HOM (+/+) vs WT (X/X), factor “genotype”: p = 0.0001, F (2, 69) = 10.31; factor “time”: p < 0.0001, F (4.897, 337.9) = 223.0; interaction not significant; *post hoc* Dunnett's test, Fig. 1E).

Tests which were scored as present or absent were compared between groups based on day of onset. Grasping reflex of hind paws showed a significant difference between Cdkl5^E364X^ HOM (+/+) females, but not HET (X/+), when compared to sex-matched WT, with homozygous individuals needing three days more (on average) that the counterparts in developing the reflex (Fig. 1M). No differences were observed among males or in the front paws reflex in all Cdkl5^E364X^ individuals (Supplementary Fig. S3A).

No differences were observed for eyes open tests in both Cdkl5^E364X^ male and female mice (see Supplementary Figs. S3C and D, respectively). Grip strength analysis achieved statistical significance (mixed-effect model, factor “genotype”, p = 0.0001, F (2, 69) = 10.55; factor “time”: p < 0.0001, F (3.889, 267.3) = 105.7; interaction: p < 0.0001, F (14, 481) = 3.823, *post hoc* Dunnett's test, Fig. 1L and M) only comparing female WT (X/X) with HET (X/+) and HOM (+/+) individuals, showing that female Cdkl5^E364X^ genotypes needed more days to develop a grip strength comparable to the one of WT sex-matched individuals (Fig. 1L). No difference was observed in males (see Supplementary Fig. S3B).

Sensory reflex and auditory startle were performed only in pups of the first litters and then suspended as it was impossible for operators to identify with certainty their day of appearance.

Motor neuron maturation was evaluated using the clasping test from PND 7 to PND 21. As previously reported in different Cdkl5 mouse models [[15], [16], [17],^19^], our results confirmed a significantly higher incidence of hindlimb clasping in male HEM compared to sex-matched WT animals (mixed-effect model with Geisser-Greenhouse correction, factor “genotype”: p = 0.0147, F (1, 77) = 6.225; factor “time”: p < 0.0001, F (2.694, 205.5) = 30.45; interaction: p = 0.0037, F (7, 534) = 3.049; *post hoc* Sidak's test, Fig. 1F), while in females statistical significance was only achieved at PND 11, 13 and 17 (two-way ANOVA, WT (X/X) vs. HOM (+/+) and HET (X/+), factor “genotype”: not significant; factor “time”: p < 0.0001, F (3.221, 231.9) = 26.99; interaction not significant; *post hoc* Dunnett's test, Fig. 1H).

The cliff aversion reflex requires the combination of tonic labyrinthine reflex, strength and coordination to move away from the drop [26]. This reflex appeared in older Cdkl5^E364X^ pups compared to their WT littermates, and the difference was statistically significant in both males (Student's t-test, HEM (+/Y) vs WT (X/Y), p = 0.0060) and females (Kruskal-Wallis test with *post hoc* Dunn's test, p = 0.0008) (Fig. 1G–I).

### 3.4 Locomotion, sensorimotor integration and gait analysis

**Fig. 2:**
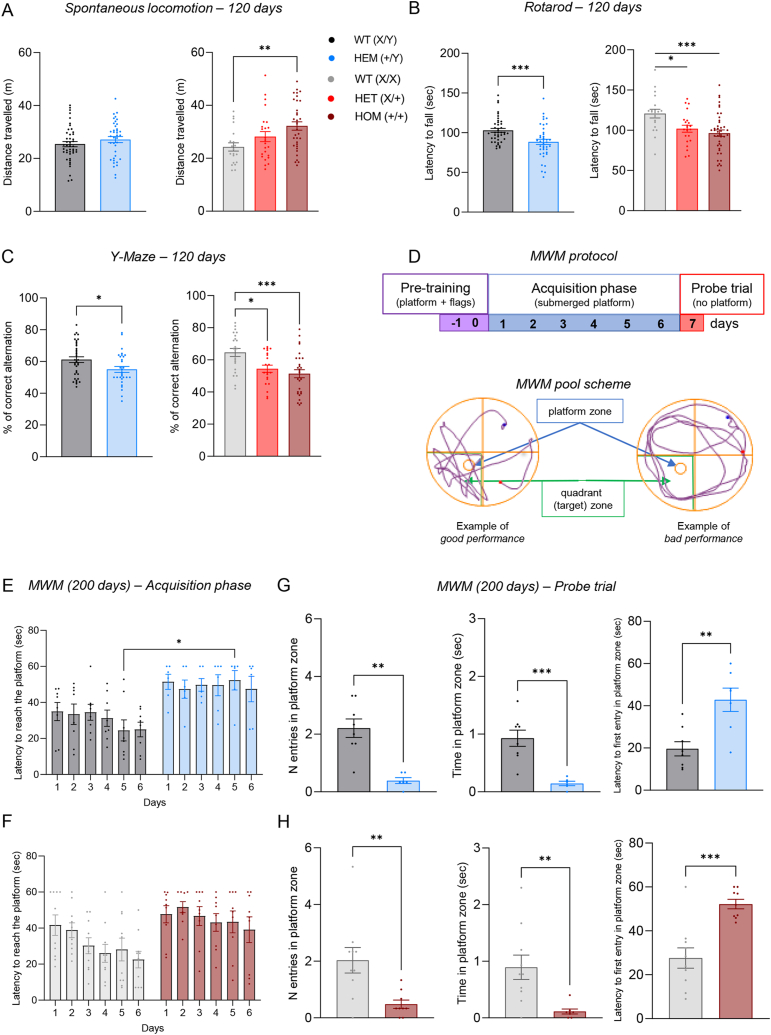
Locomotion, sensorimotor coordination, learning and memory (A) Spontaneous locomotion in an open-field arena in all genotypes in male and female mice, expressed as distance traveled. Statistical analysis showed no significance between male genotypes (HEM (+/Y), n = 38; WT (X/Y), n = 45), while female HOM (+/+) (n = 34) showed a significantly higher activity than sex-matched WT (n = 20) (p = 0.0051); no difference was observed between HET (X/+) (n = 24) vs WT and vs HOM (for statistical analysis values, see Results section; asterisks represent the multiple comparison with Tukey's test). (B) Sensorimotor coordination in the rotarod test in all genotypes in male and female mice, expressed as latency to fall. Males, asterisks represent the results of Mann-Whitney test on WT (X/Y) (n = 41) vs. HEM (+/Y) (n = 40); females, asterisks represent the results of multiple comparisons one-way ANOVA with Tukey's test on WT (X/X) (n = 19) vs. HET (X/+) (n = 22) (p = 0.0371) and HOM (+/+) (n = 41) (p = 0.0013) (for statistical analysis values, see Results section). (C) Spatial memory evaluated by Y-maze test in male and female mice, expressed as % of correct alternations. Males, asterisks represent the results of Student's t-test on WT (X/Y) (n = 36) vs. HEM (+/Y) (n = 30); females, asterisks represent the results of multiple comparisons one-way ANOVA with *post hoc* Tukey's test on WT (X/X) (n = 23) vs. HET (X/+) (n = 21) (p = 0.0186) and HOM (+/+) (n = 28) (p = 0.0006). For statistical analysis values, see Results section. (D) Experimental protocol of the Morris water maze test: violet, pre-training test; blue, acquisition phase; red, probe trial. Schematic representation of the MWM pool, with example of a good performance trace and a bad performance one; the platform zone (blue) and the quadrant (or target) zone (green) are indicated. (E, F, G, H) Spatial and long-term memory evaluated by Morris water maze (MWM) in male and female Cdkl5^E364X^ mice. (E, F) Graphs show the acquisition phase of the test in males (E) and females (F), which measures the latency to reach the platform; (G, H) graphs show the selected parameters of the probe trial for males (G) and females (H), which are the number of entries in platform zone, the time spent in platform zone and the latency to the first entry in platform zone.Acquisition phase of males, the asterisk represents the result of multiple comparison two-way ANOVA with *post hoc* Sidak's test in WT (X/Y) (n = 8) vs. HEM (+/Y) (n = 6) (multiple comparison for the factor “genotype”, day 5, WT (X/Y) vs HEM (+/Y), p = 0.0258); acquisition phase of females, no significant comparisons (two-way ANOVA with *post hoc* Sidak's test) in WT (X/X) (n = 10) vs. HOM (+/+) (n = 9). Probe trial, number of entries in platform zone: for both males and females, the asterisks represent the results of Mann-Whitney test in WT (X/Y) (n = 8) vs. HEM (+/Y) (n = 6) and WT (X/X) (n = 10) vs. HOM (+/+) (n = 9), respectively. Probe trial, time in platform zone: the asterisks represent the results of Student's t-test in males WT (X/Y) (n = 8) vs. HEM (+/Y) (n = 6) and of Mann-Whitney test in female WT (X/X) (n = 10) vs. HOM (+/+) (n = 8). Probe trial, latency to first entry in platform zone: for both males and females, the asterisks represent the results of Student's t-test in males WT (X/Y) (n = 8) vs. HEM (+/Y) (n = 7) and females WT (X/X) (n = 10) vs. HOM (+/+) (n = 9), respectively.For statistical analysis values, see Results section.∗p < 0.05; ∗∗p < 0.005; ∗∗∗p < 0.001; ∗∗∗∗p < 0.0001.All data are expressed as mean ± SEM.

The open field test to assess spontaneous locomotor activity highlighted a trend toward hyperactivity in Cdkl5^E364X^ mice compared to their same-sex WT counterparts at 120 days (males: Student's t-test, not significant; females: one-way ANOVA, p = 0.0066, F = 5.367, *post hoc* Tukey's test), which reached statistical significance in the comparison between HOM (+/+) and WT (X/X) females (p = 0.0051, Fig. 2A). The same trend was already present at 80 days of age (see Supplementary Figs. S4A and B). These results are in line with those reported for different Cdkl5 mouse models [[15], [16], [17],^19^,^21^]. Similarly, analysis of motor coordination using the rotarod test evidenced a significantly lower latency to fall in Cdkl5^E364X^ animals at 120 days of age compared to their WT same-sex counterparts (males, Mann-Whitney test, HEM (+/Y) vs WT (X/Y), p = 0.0005; females, one-way ANOVA, p = 0.0019, F = 6.778, *post hoc* Tukey's test, Fig. 2B), as previously reported [15,17,19,21,31,38]. At 80 days of age, no significant differences were present in both males and heterozygous females when compared to sex-matched WT, while in homozygous female the impairment is already present (see Supplementary Figs. S4C and D).

Computerized gait analysis using CatWalk showed a significant alteration in Cdkl5^E364X^ mice compared to WT in kinetic parameters, whereas no differences were observed in coordination and paw spatial parameters (see Table 4 for analysis at 80 days; Supplementary Table S3 for analysis at 120 days).

**Table 4:** Gait analysis performed at 80 days with Catwalk apparatus.

			Males	Females		
			WT (X/Y), n = 43 vs. HEM (+/Y), n = 39; p	WT (n = 18) vs HET (n = 24); p	WT (n = 18) vs HOM (n = 41); p	Overall test, p
**Spatial values**	**Paw area**	**Front**	0.4297 (Mann Whitney test)	0.3618	0.2780	0.2920 (Kruskal-Wallis test)
				(*post hoc* Dunn's test)		
		**Hind**	0.6753 (Mann Whitney test)	0.3345	0.3345	0.3852 (One Way ANOVA)
				(*post hoc* Holm-Sidak's test)		
**Kinetic values**	**Base of Support (BOS)**	**Front**	**0.0340** (Student *t*-test)	0.6259	**0.0040**	**0.0068** (Kruskal-Wallis test)
				(*post hoc* Dunn's test)		
		**Hind**	**<0.0001** (Student *t*-test)	**0.0202**	**0.0011**	**0.0023** (One Way ANOVA)
				(*post hoc* Holm-Sidak's test		
	**Stride length**	**Front**	**0.0018** (Student *t*-test)	**0.0008**	**0.0001**	**0.0001** (Kruskal-Wallis test)
				(*post hoc* Dunn's test)		
		**Hind**	**0.0022** (Student *t*-test)	**<0.0001**	**<0.0001**	**<0,0001** (One Way ANOVA)
				(*post hoc* Holm-Sidak's test)		
	**Duty Cycle (%)**	**Front**	0.3124 (Mann Whitney test)	0.9525	**0.0444**	**0.0042** (One Way ANOVA)
				(*post hoc* Tukey's test)		
		**Hind**	**0.0029** (Mann Whitney test)	**0.0327**	**0.0002**	**0.0006** (Kruskal-Wallis test
				(*post-hoc* Kruskal-Wallis test)		
**Coordination**	**Regulatory Index (%)**		0.4287 (Mann Whitney test)	0.7463	0.3428	0.3920 (Kruskal-Wallis test)
				(*post hoc* Dunn's test)		
	**Cadence**		0.6162 (Student *t*-test)	0.0977	0.0646	0.0770 (Kruskal-Wallis test)
				(*post hoc* Dunn's test)		

Six parameters are reported. The Table summarizes the p values of comparisons between Cdkl5 mice and sex-matched WT (statistical analysis details are reported, bold highlights p < 0.05 which is considered significant).

In particular, BOS parameter, stride length and hindlimb duty cycle were significantly affected by the presence of the Cdkl5^E364X^ variant in both males and females compared to same-sex WT individuals. The front limb duty cycle was significantly altered in female HOM (X/X) when compared to sex-matched WT. None of the other parameters analyzed was altered in Cdkl5^E364X^ mice compared to the respective WT controls. These results are in line with the previously reported mild gait impairment in Cdkl5 mouse models [31,39].

### 3.5 Learning and memory

Impairment in hippocampal-dependent working memory has been widely assessed in different *Cdkl5* mouse models [15,19,21,35,39], and our Y-maze results are in line with these findings, highlighting a significant change in the percentage of correct alternations at 120 days of age in both male (HEM (+/Y) vs WT (X/Y), Student's t-test, p = 0.0208) and female (HET (X/+) and HOM (+/+) vs WT (X/X), one-way ANOVA, p = 0.0007, F = 8.050, post hoc Tukey's test) *Cdkl5*^*E364X*^ mice compared with same-sex WT animals (Fig. 2C). The same trend was already present at 80 days of age (see Supplementary Figs. S4E and F). The number of entries reached statistical significance only in the comparison between female HOM (+/+) and sex-matched WT (see Supplementary Figs. S5A and B).

Considering the results obtained to this point in the characterization of our adult Cdkl5 model with spontaneous locomotion, rotarod and Y-maze, where no differences were observed between female HET (X/+) and HOM (X/X), from this point on we proceeded with the remaining characterizations only in female HOM (X/X).

The spatial memory and cognitive profile of *Cdkl5*^*E364X*^ mice was further evaluated using the Morris water maze test (MWM) (Fig. 2D). The pre-training phase of the test, characterized by the presence of flags on the platform, was used to exclude any visual impairment in CDD mice, as an improvement in the performance is present in both homozygous females (which is statistically significant on both day 1 and day 2) and hemizygous males (only a trend is visible, but it does not reach significance) (see Supplementary Figs. S5C and D). Mice carrying the pathological variant showed a significant impairment in memory and cognition when compared to sex-matched WT, as shown by different test parameters, namely a reduced number of entries in platform zone (males, HEM (+/Y) vs WT (X/Y), Mann-Whitney test, p = 0.0017, Fig. 2G; females, HOM (+/+) vs WT (X/X), Mann-Whitney test, p = 0.0039, Fig. 2H), reduced time in platform zone (males, HEM (+/Y) vs WT (X/Y), Student's t-test, p = 0.0005, Fig. 2G; females, HOM (+/+) vs WT (X/X), Mann-Whitney test, p = 0.0027, Fig. 2H), and an increased latency to the first entry in platform zone (males, HEM (+/Y) vs WT (X/Y), Student's t-test, p = 0.0028, Fig. 2G; females, HOM (+/+) vs WT (X/X), Student's t-test, p = 0.0003, Fig. 2H). No significant differences were observed among groups for the time in target (quadrant) zone (see Supplementary Figs. S5E and F). These findings are in line with the results obtained previously [38] and with those obtained using the Barnes maze [39], an alternative to the MWM.

The impairment of *Cdkl5*^*E364X*^ mice in learning and memory was already evident during the six days of acquisition tests which preceded the probe trial. In this phase, the platform was still visible, revealing a significant learning impairment in both male and female *Cdkl5*^*E364X*^ mice compared to same-sex WT animals (males HEM (+/Y) vs WT (X/Y), two-way ANOVA, factor “genotype”: p = 0.0011, F (1, 12) = 18.03; factor “time” and interaction not significant, *post hoc* Sidak's test, Fig. 2E; females, HOM (+/+) vs WT (X/X), two-way ANOVA, factor “genotype”: p = 0.0040, F (1, 17) = 11.03; factor “time”: p = 0.0303, F (3.431, 58.33) = 3.035; interaction not significant; *post hoc* Sidak's test, Fig. 2F).

### 3.6 Cdkl5 gene expression is reduced in the CNS but not in peripheral tissues

**Fig. 3:**
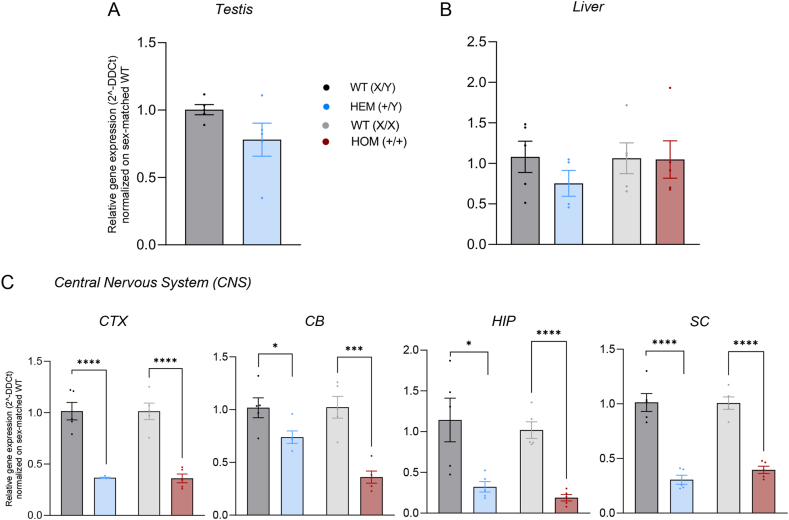
Expression of the murine *Cdkl5* gene in peripheral tissues and in the central nervous system The graphs show *Cdkl5* expression in (A) the testis of male WT (X/Y) (n = 5) and HEM (+/Y) (n = 5) mice; (B) the liver of male WT (X/Y) (n = 5) and HEM (+/Y) (n = 4), and female WT (X/X) (n = 5) and HOM (+/+) (n = 5) animals; (C) different areas of the CNS in male WT (X/Y) and HEM (+/Y), and female WT (X/X) and HOM (+/+) mice (five animals per group were included in the analysis). Student's t-test was performed between *Cdkl5*^*E364X*^ and WT animals of the same sex and asterisks represent the related statistical significance (∗p < 0.05, ∗∗∗p < 0.001, ∗∗∗∗p < 0.0001). For statistical analysis values, see Results section. Data are represented as mean ± SEM. Relative gene expression was calculated using 2^−ΔΔCT^ method using the WT of each sex as control for each tissue of interest.Abbreviations: CTX, cerebral cortex; CB, cerebellum; HIP, hippocampus; SC, spinal cord; CNS, central nervous system.

As described in the Methods section, we analyzed the expression of the murine *Cdkl5* gene in four different CNS areas, namely the cerebral cortex (CTX), cerebellum (CB), hippocampus (HIP) and spinal cord (SC), and in two peripheral tissues where *CDKL5* gene expression in humans is described as high (testicles) and low (liver), respectively [40,41]. The presence of the humanized gene insertion containing the loss-of-function *Cdkl5*^*E364X*^ mutation did not result in an alteration of gene expression in either testis (Fig. 3A) or liver (Fig. 3B). Firstly, we compared the analysis of *Cdkl5* gene expression between testis of male WT and liver and CNS (CTX) of male and female WT animals, normalizing on the expression in male WT testis, confirming that the testis and CNS are high-expressing tissues in mice as well as in humans. We additionally confirmed the liver to be low-expressing in mice also: this tissue also shows a significant sex-related difference, with females expressing a higher level of the *Cdkl5* gene (fold change: +2.68) compared to males (Supplementary Fig. S1D). For the analysis of *Cdkl5* gene expression in the different areas of CNS, we compared the data obtained between Cdkl5 and WT animals of the same sex, and we did observe a significant reduction in *Cdkl5* gene expression in all the CNS areas considered, both in males and females (Student's t-test, CTX, males, HEM (+/Y) vs WT (X/Y), p < 0.0001; females, HOM (+/+) vs WT (X/X), p < 0.0001; CB, males, HEM (+/Y) vs WT (X/Y), p = 0.0366; females, HOM (+/+) vs WT (X/X), p = 0.0005; HIP, males, HEM (+/Y) vs WT (X/Y), p = 0.0177; females, HOM (+/+) vs WT (X/X), p < 0.0001; SC, males, HEM (+/Y) vs WT (X/Y), p < 0.0001; females, HOM (+/+) vs WT (X/X), p < 0.0001) (Fig. 3C). Moreover, no difference was observed between WT male and female individuals (see Supplementary Fig. S6A).

### 3.7 The Cdkl5 p.E364X mutation affects the CDKL5 protein expression in mouse brains

**Fig. 4:**
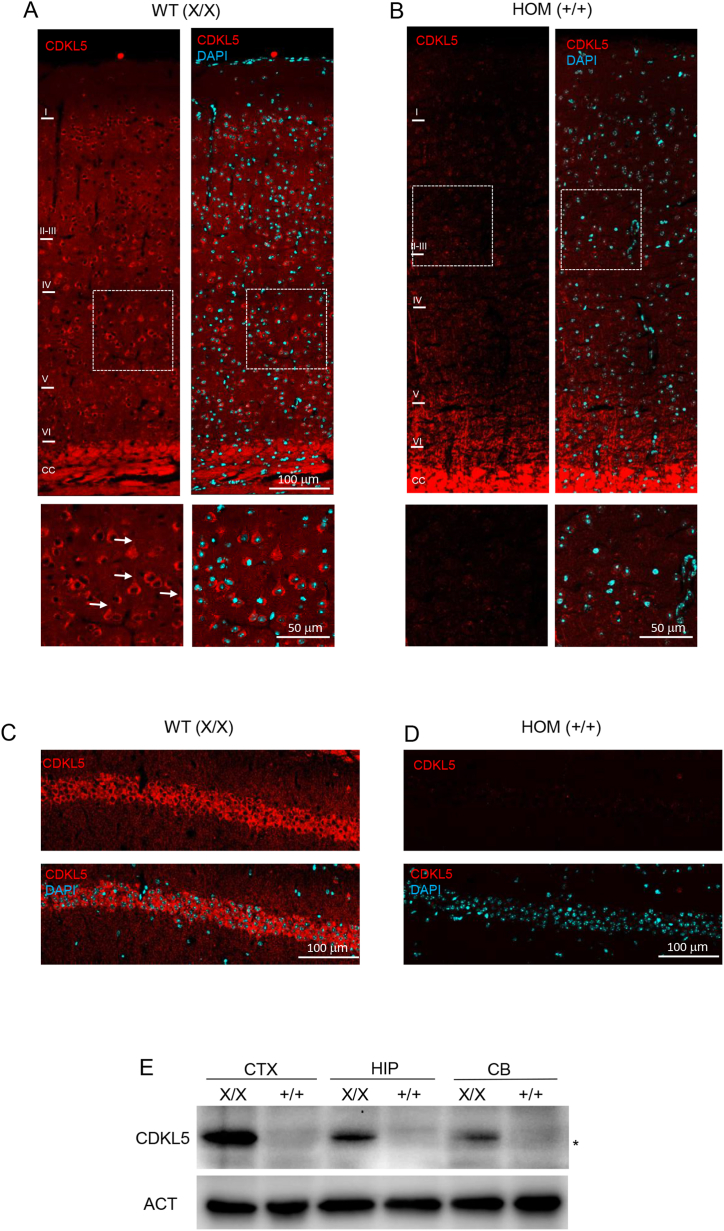
The p.E364X mutation abolishes the protein expression of CDKL5 in female HOM (+/+) brain (A–D) Representative low magnification images showing CDKL5 (red) immunofluorescence and DAPI staining (cyan) throughout the layers of the S1 cortex (A, B) and in the CA1 region of the hippocampus (C, D) of both WT (X/X) and HOM (+/+) female mice. (A1-B1). An unspecific secondary fluorescence antibody staining of myelinated fibers in the corpus callosum (CC) is visible (A, B). Higher magnifications of the boxed areas in A and B are shown (below) and arrows point to immunostained apical dendrites (proximal portion). (E) Western blotting showing the expression of both CDKL5 and actin in total lysates of cortex, hippocampus and cerebellum from both WT (X/X) and HOM (+/+) female mice. Asterisks indicate an unspecific band detected by the anti-CDKL5 antibody. Abbreviations: *CTX, cerebral cortex; HIP, hippocampus; CB, cerebellum*.

To confirm that the pathogenic Cdkl5^E364X^ variant affects protein expression, we performed both histological and biochemical analyses. Immunofluorescence using an anti-CDKL5 sheep antibody decorated both the cell body and the proximal portion of the apical dendrite of pyramidal neurons across all cortical layers in S1 (Fig. 4A), and the cell body of CA1 pyramidal neurons in the hippocampus (Fig. 4C) of WT female mice, a finding consistent with previous observations [42]. As expected, no CDKL5 immunosignal was detectable in comparable sections from *Cdkl5*^*E364X*^ HOM (+/+) female mice (Fig. 4B–D). The staining of myelinated fibers visible in the corpus callosum (Fig. 4A and B) resulted from an unspecific myelinated fibers labelling of the anti-sheep secondary fluorescent antibody. Western blot analyses on cerebral cortex, hippocampus and cerebellum lysates also confirmed the complete absence of CDKL5 expression in female mutant animals (Fig. 4E). Interestingly, this analysis confirmed previous observations showing that CDKL5 expression is higher in the cerebral cortex compared to other brain regions [42]. In summary, these findings suggest that the E364X mutation completely abolishes the expression of CDKL5 in the brain.

### 3.8 The Cdkl5 ^E364X^ variant causes a modification in CNS cell population and alters the GABA pathway

**Fig. 5:**
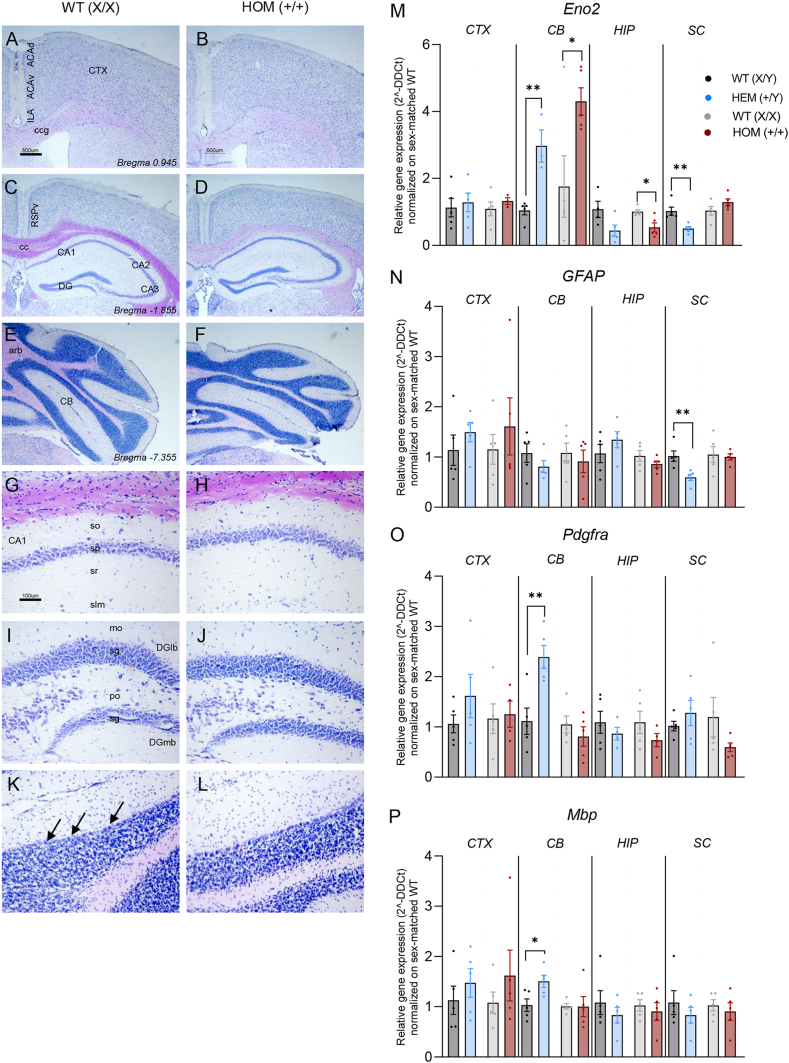
Histological analysis and cell composition of brain areas in CDKL5^E364X^-mutated and C57BL6 WT mice (A–F) Low-power micrographs of toluidine blue-stained sections, showing the cerebral cortex (A, B), hippocampus (C, D) and cerebellum (E, F) in female WT (X/X) (A, C, E) and HOM (+/+) (B, D, F) mice. (G, L) High-power micrographs of toluidine blue-stained sections, showing the CA1 hippocampal field (G, H), dentate gyrus (I, J), and cerebellar cortex (K, L). Arrows in (K) show Purkinje cells. (M − P) Graphs show the gene expression of lineage-specific genes representing neurons (*Eno2*, M), astrocytes (*Gfap*, N), oligodendrocyte precursor cells (*Pdgfra*, O), and myelinating oligodendrocytes (*Mbp*, P) in different areas of the CNS (separated by a black continuous vertical line) of male WT (X/Y) and HEM (+/Y) and female WT (X/X) and HOM (+/+) animals. Student's t-test was performed between *Cdkl5*^*E364X*^ and WT animals of the same sex, and asterisks represent the related statistical significance among the sexes (male, female) separated by a grey dotted vertical line (∗p < 0.05, ∗∗∗p < 0.001, ∗∗∗∗p < 0.0001). For statistical analysis values, see Results section. Data are represented as mean ± SEM. Relative gene expression was calculated using 2-ΔΔCT method normalizing on the WT of each sex for each tissue of interest. Number of animals included in the analysis:Eno2 in CTX, males WT (X/Y) n = 5, HET (+/Y) n = 5; females, WT (X/X) n = 5, HOM (X/X) n = 3.Eno2 in CB, males WT (X/Y) n = 5, HET (+/Y) n = 3; females, WT (X/X) n = 5, HOM (X/X) n = 5.Eno2 in HIP, males WT (X/Y) n = 4, HET (+/Y) n = 5; females, WT (X/X) n = 5, HOM (X/X) n = 5.Eno2 in SC, males WT (X/Y) n = 5, HET (+/Y) n = 5; females, WT (X/X) n = 5, HOM (X/X) n = 5.GFAP, five animals were included in the analysis for each group in all tissues and genes tested.Pdgfra, CTX, males WT (X/Y) n = 5, HET (+/Y) n = 5; females, WT (X/X) n = 5, HOM (X/X) n = 4.Pdgfra in CB, males WT (X/Y) n = 5, HET (+/Y) n = 5; females, WT (X/X) n = 5, HOM (X/X) n = 5.Pdgfra in HIP, males WT (X/Y) n = 5, HET (+/Y) n = 4; females, WT (X/X) n = 5, HOM (X/X) n = 4.Pdgfra in SC, males WT (X/Y) n = 5, HET (+/Y) n = 5; females, WT (X/X) n = 5, HOM (X/X) n = 5.Mbp, five animals were included in the analysis for each group in all tissues and genes tested.Abbreviations: ACAv, anterior cingulate area, ventral part; ACAd, anterior cingulate area, dorsal part; Arb, arbor vitae; cc, corpus callosum; CA1, hippocampus, CA1 field; CA2, hippocampus, CA2 field; CB, cerebellum; Ccg, genu of corpus callosum; CP, caudoputamen; CTX, cerebral cortex; DG, hippocampus, dentate gyrus; DGlb, DG, lateral blade; DGmb, DG, medial blade; Eno2, enolase 2; Gfap, glial fibrillary acidic protein; HIP, hippocampus; ILA, infralimbic area; Mbp, myelin basic protein; mo, DG, molecular layer; Pdgfra, platelet derived growth factor receptor alpha; po, DG, polymorph layer; RSPv, retrosplenial area, ventral part; SC, spinal cord; sg, DG, granule cell layer; slm, CA1, stratum lacunosum-moleculare; so, CA1, stratum oriens; sp, CA1, pyramidal layer; sr, CA1, stratum radiatum.

We performed an observational structural study of the brain in WT and HOM (+/+) female mice, using toluidine blue-stained coronal sections, as shown in Fig. 5. Coronal sections collected at rostrocaudal levels 0.945, −1.855 and −7.355 are shown at low (A-F) and high (G-L) magnification. Micrographs show cortical areas (cerebral, hippocampal and cerebellar) where no gross differences between genotypes were observed.

To investigate the impact of the pathogenic *Cdkl5*^*E364X*^ variant on cell populations in the different CNS areas (i.e., CTX, CB, HIP, and SC), qPCR analysis was performed to quantify the expression of neural lineage-specific genes (i.e., *Eno2* for neurons, *Gfap* for astrocytes, *Pdgfra* for OPCs, and *Mbp* for matu

re myelinating oligodendrocytes) (Fig. 5M − P).

Neuronal populations were significantly altered by the *Cdkl5*^*E364X*^ variant in different CNS areas. Compared to WT animals of the same sex, *Eno2* expression in the cerebellum was increased in both male HEM (+/Y) and female HOM animals (+/+) (Student's t-test, p = 0.0027, p = 0.0348, respectively). In the hippocampus, only female HOM (+/+) animals showed a reduction in *Eno2* expression compared to female WT mice (Student's t-test, p = 0.0132), mirroring the expression changes of the same gene in the spinal cord in male HEM animals (+/Y) (Student's t-test, p = 0.0048) (Fig. 5M).

The *Cdkl5*^*E364X*^ variant also significantly affected both astrocyte and oligodendroglial lineages in male HEM (+/Y) compared to male WT animals. In particular, *Gfap* gene expression was reduced in the spinal cord (Student's t-test, p = 0.0078) (Fig. 5N), whereas expression of both the OPC-related gene *Pdgfra* and the mature oligodendrocyte-related gene *Mbp* was increased in the cerebellum (Student's t-test, p = 0.0063, p = 0.0268, respectively) (Fig. 5O and P). As for *Cdkl5* gene expression, also for the population-specific genes analyzed no differences were observed between WT males and females (see Supplementary Figs. S6B, C, D, E).

The results of molecular analysis, together with the rotarod functional test outcome, highlighted the cerebellum as a CNS area highly influenced by the presence of our pathogenic variant in *Cdkl5* gene, confirming a previous study on a *Cdkl5* KO mouse model in which we showed an altered ratio between glutamatergic and GABAergic synapse markers in this area [31]. Moreover, alterations in hippocampal structure and functionality, such as in hippocampus-dependent behaviors as also observed in the *Cdkl5*^*E364X*^ mouse, are distinctive of CDD mouse models [43], therefore we selected both the cerebellum and the hippocampus for a more in-depth characterization of the synaptic plasticity molecular machinery.

Using PCR array technology, we analyzed 84 genes involved in synaptic plasticity pathways in the hippocampus and cerebellum of both male WT (X/Y) and HEM (+/Y) and female WT (X/X) and HOM (+/+) animals. The array comprised immediate-early genes, late response genes, long-term potentiation genes, long-term depression genes, cell adhesion molecules, extracellular matrix molecules, CREB cofactors, neuronal receptors and postsynaptic density complexes, as well as the five most common housekeeping genes (see full list of genes in Supplementary Table S1).

**Fig. 6:**
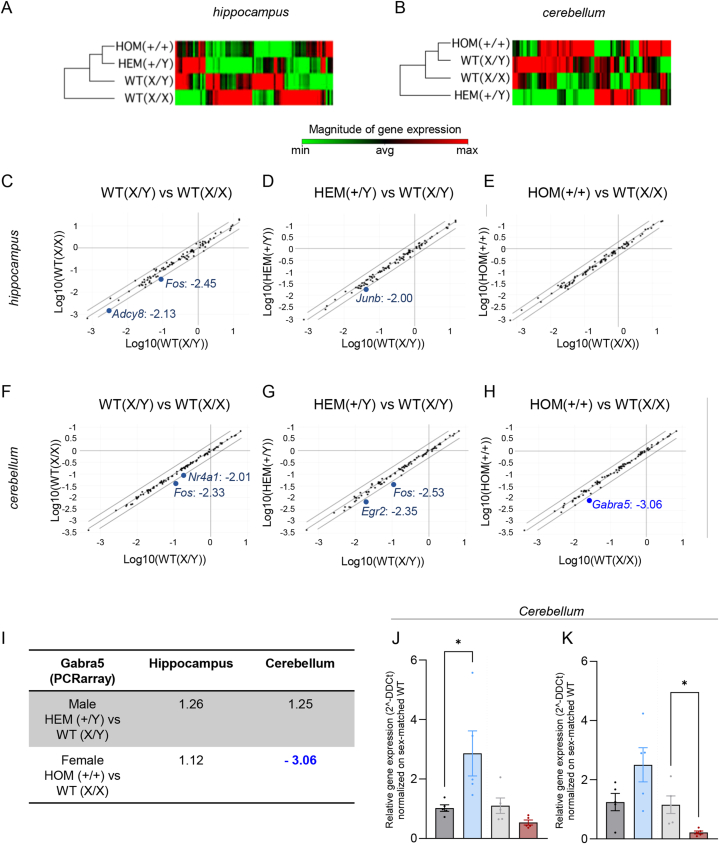
PCR array analysis and validation of genes involved in synaptic plasticity processes (A–B) Cluster analysis of the PCR array results on 84 genes involved in synaptic plasticity in the hippocampus (A) and cerebellum (B). The magnitude of expression of each gene across the four analyzed groups is indicated via a reference color scale (minimum, green; maximum, red). Clusterization analysis was performed on the basis of expression pattern similarity. (C–E) Scatter plots of all PCR array results in the hippocampus, analyzed as the relative expression of male WT (X/Y) compared to female WT (X/X) animals (C), male HEM (+/Y) compared to WT (X/Y) animals (D), and female HOM (+/+) compared to WT (X/X) animals (E). Only genes with a fold change >2 are indicated. (F–H) Scatter plots of the entire PCR array in the cerebellum, analyzed as the relative expression of male WT (X/Y) compared to female WT (X/X) animals (F), male HEM (+/Y) compared to WT (X/Y) animals (G), and female HOM (+/+) compared to WT (X/X) animals (H). Only genes with a fold change >2 are indicated. (I) Values of fold change for the *Gabra5* gene expression in the hippocampus and cerebellum between the analyzed groups. (J–K) Relative expression of *Gabra1* (J) and *Gabra5* (K) in male HEM (+/Y) compared to WT (X/Y) and female HOM (+/+) compared to WT (X/X) animals. Student's t-test was performed between *Cdkl5*^*E364X*^ and WT animals of the same sex and asterisks represent the related statistical significance among the sexes (male, female) separated by a grey dotted vertical line (∗p < 0.05, ∗∗∗p < 0.001, ∗∗∗∗p < 0.0001). For statistical analysis values, see Results section. Five animals per group were pooled for the PCR array analysis (A–I), while five animals per group as single samples were included in the qPCR analysis (J–K). qPCR analysis data are shown as a mean value ± SEM, and relative gene expression was calculated using 2-ΔΔCT method normalizing on the WT of each sex.Abbreviations: Adcy8, adenylate cyclase 8; Egr2, early growth response 2; Fos, FBJ osteosarcoma oncogene; Gabra1, gamma-aminobutyric acid (GABA) A receptor, subunit alpha 1; Gabra5, gamma-aminobutyric acid (GABA) A receptor, subunit alpha 5; Junb, Jun-B oncogene; Nr4a1, nuclear receptor subfamily 4, group A, member 1.

Cluster analysis was performed on the expression levels of the gene set analyzed in the different genotypes. In the hippocampus, the closest groups were the two comprising the mutated female HOM (+/+) and male HEM (+/Y) animals, which share the most similar expression pattern to both male and female WT mice (Fig. 6A; full cluster analysis image in Supplementary Fig. S7). The cerebellum, on the other hand, showed no recognizable sex- or genotype-related clusterization pattern, as the groups expressing the *Cdkl5*^*E364X*^ variant were localized at the extremities of the clustergram (Fig. 6B; full cluster analysis image in Supplementary Fig. S8).

We then performed a comparative analysis of the gene expression levels in the cerebellum and the hippocampus, using a two-fold change value as a cut-off for significant changes. We initially compared the gene set expression between male and female WT mice, finding a significant reduction, albeit below a fold change of 2.5, in the expression levels of two genes in males compared to females - *Fos* (FBJ osteosarcoma oncogene) and *Adcy8* (Adenylate cyclase 8) (Fig. 6C). We then compared the gene set expression levels between the animals expressing the *Cdkl5*^*E364X*^ variant and the relative WT in both males (Fig. 6D) and females (Fig. 6E). No significant variations were detected, apart from *Junb* (Jun-B oncogene) which was at the threshold limit, being two-fold less expressed in HEM (+/Y) than in WT (X/Y) animals.

The same analysis was performed for the cerebellum gene expression profile. The two WT groups, males and females, again showed two genes less expressed in male WT (X/Y) mice, *Fos* and *Nr4a1* (nuclear receptor subfamily 4, group A, member 1), with a fold change below 2.50 (Fig. 6F).

In males, the presence of the *Cdkl5*^*E364X*^ variant resulted in a reduction of *Fos* and *Egr2* (early growth response 2) expression levels, albeit with a fold change at the limit of the cut-off value (Fig. 6G). In females, only *Gabra5* (gamma-aminobutyric acid A receptor, subunit alpha 5) was differentially expressed, with a fold change >3 (3.06 times less in the HOM (+/+) compared to WT (X/X)) (Fig. 6H). In particular, the expression of *Gabra5* was unaffected by the presence of the pathogenic variant in the male cerebellum and in both groups in the hippocampus (Fig. 6I), suggesting a specific regulation in the female cerebellum.

To validate the PCR array results obtained from pooled samples, we performed qPCR analysis on individual samples to better characterize the expression of genes involved in GABA synthesis (*Gad65* and *Gad67*), transport (*Gat1* and *Vgat*) and action (*Gabra1* and *Gabra5*). No differences were detected for genes involved in synthesis or transport (data are available in the repository linked in the dedicated section of the manuscript), while the expression of the two GABA receptors, *Gabra1* and *Gabra5*, was significantly affected by the presence of the *Cdkl5*^*E364X*^ variant. In particular, *Gabra1* expression showed contrasting trends depending on the sex of the animal, being significantly increased in male HEM (+/Y) compared to WT (X/Y) (Student's t-test, p = 0.0429) and showing a trend towards reduced in female HOM (+/+) compared to WT animals (X/X), that did not reach statistical significance (Fig. 6J). On the other hand, we confirmed the absence of significant *Gabra5* expression changes in males, and most importantly, a significantly reduced expression in female HOM (+/+) compared to WT mice (X/X) (Student's t-test, p = 0.0148) (Fig. 6K).

## 4 Discussion

In this study we present a new mouse model for CDKL5 disease, which has been designed and characterized with the goal of evaluating the suitability of this model as testing tool for personalized gene therapy testing. Accordingly, we generated a CDD mouse model humanized for the presence of a well-characterized nonsense variant found in the C-terminal domain of CDKL5 protein of human patients. The p.E364X pathogenic variant is located in the C-terminal domain of the kinase, therefore, similarly to the other pathogenic variants described in patients in the aminoacidic region 172–781 [3], it is associated with a milder phenotype. In line with best practices in the characterization of novel transgenic colonies, we characterized both male (wild type and hemizygous) and female (wild type, heterozygous and homozygous) mice. In particular, for the female Cdkl5^E364X^ mice, the assessment of neuromaturation up to 21 postnatal days and a battery of neurobehavioral test in adult life (spontaneous locomotion, rotarod and Y-maze) were performed on both heterozygous and homozygous mice compared to wild type. Given the absence of significant difference between adult performance of heterozygous and homozygous females, the second and more demanding battery of tests, comprising MWM, molecular and immunofluorescence characterization, was performed only on homozygous individuals, in order to avoid the possible bias due to the intrinsic heterogeneity associated with heterozygosity and thus unveil, if present, a link between the behavioral and the molecular phenotype associated to the mutation of interest. This mouse model presents with severe neurological and neurobehavioral phenotypes affecting locomotion and cognitive domains, during both neurological maturation and in adulthood. Molecular data suggest an imbalance in GABAergic pathways in the cerebellum and in other brain areas known to be involved in seizures.

The International CDKL5 Disorder Database provided a large body of information on CDD patients, allowing explorative studies of phenotype/genotype matching [13]. Although no clear correlation with variant type (missense vs nonsense) or position within the protein have been observed, authors have reported differences in the severity of phenotype in individuals with different *CDKL5* variants, highlighting how variants in close proximity on the *CDKL5* gene can result in widely varying clinical phenotypes, mostly evaluated on the basis of achievement of developmental milestones, motor and communication ability and epilepsy burden. In patients, pathogenic truncating variants have been described throughout the entire coding sequence of the *CDKL5* gene, with more than 200 pathogenic variants described in 285 patients. The first attempt to categorize pathogenic variants, with the aim of identifying related phenotypic traits, involved the distribution of pathogenic variants into four groups according to their position on the gene and the associated functional consequences [3].

As historically known, knockout mouse models, generated through the insertion of null mutations in the gene/s of interest, are an invaluable tool for the understanding of the role/s of specific loci, with the major limitations being the modeling of loss of function, but not gain of function, disease-related triggers, and the study of the overall loss of the target gene-derived protein, but not the fine investigation of the consequences of specific pathogenic variants, often described in disease driver genes and responsible for the phenotypic heterogeneity observed in patients. On the other hand, the generation of knockin mouse models, thanks to the most recent genetic engineering technologies like CRISPR/Cas9, allows the site-specific insertion of patient-derived pathogenic variants, enabling the investigation of the possibly related heterogeneity [44]. Therefore, the choice between opting for a knockout or a knockin mouse model depends on the scientific question of the project, with knockin models being more adequate to mimic the complex molecular panorama behind patients’-described phenotypic heterogeneity and then representing a more suitable tool for therapeutic efficacy studies. Most importantly, knockin technology allows for the generation of humanized mouse models, which is the most relevant aspect when looking for potential gene therapy testing platforms. We therefore generated a new mouse model carrying a patient-derived knockin of a pathogenic variant localized in the C-terminus region of the protein, the p.E364X, to unveil potential genotype-phenotype links arising from a CDKL5 domain that, to our knowledge, has not yet been modeled. Previous studies have involved the generation of mouse models carrying a knockout mutation in the first exons of the protein (exons 2 to 6) or a site-specific knockin in the N-terminal region mimicking the truncating p.R59X pathogenic variant described in patients. Although pathogenic variants tend to be clustered in the N-terminal region of the CDKL5 protein, corresponding to the catalytic domain of the kinase, the C-terminal tail is known to be required for the subcellular distribution of CDKL5 [42]. In addition, the pathogenic C-terminus CDKL5 p.E364X variant has been characterized as the least permissive to a premature termination codon read-through therapeutic approach among a group of CDKL5 variants, possibly due to the type of termination codon generated (i.e., UAA) [45], making it refractory to the very few therapeutic options available.

With the emergence of gene editing as a promising therapeutic option, developing a model where the correction tool can be used in patients and tested without significant modifications is essential to increase the likelihood of result transferability. To achieve this aim, we generated the humanized Cdkl5^E364X^ mouse model in which, in addition to the patient-specific mutation, a 46 bp sequence around the mutation has been humanized, converting the mouse DNA to a human DNA sequence. This will allow employment of the same sgRNA for correction in both patient cells and the animal model, extremely important given the impact of the nucleotide sequence on the efficiency and specificity of the sgRNA in binding to its target sequence [46]. In particular, it has been demonstrated that both the % of G/C and the nucleotide in specific positions along the sgRNA can affect both the strength of the interaction of the sgRNA with the complementary genomic region and potentially the nuclease activity of different Cas9 enzymes [46]. As a consequence, even a single nucleotide difference in the sgRNA sequence can have a profound impact on editing efficiency, hampering the comparability of editing results between animal and human models, and consequently the transferability of results for future clinical trials in patients.

The Cdkl5 protein is absent in this model, indicating that this pathogenic variant leads to the formation of a stop codon responsible for the degradation of the entire protein, as reported in previously described CDD mice models [7]. However, Cdkl5^E364X^ mice showed an unexpected differential effect of the mutation on Cdkl5 mRNA expression in peripheral and CNS tissues. As described in the Methods section, the Cdkl5 gene expression strategy was designed to quantify the mouse mRNA expression independently of the presence of the humanized gene insert containing the mutation. Unexpectedly, both male and female Cdkl5^E364X^ mice showed a reduced expression of the Cdkl5 transcript restricted to the analyzed CNS areas (CTX, CB, HIP and SC) compared to WT littermates, while no differences were observed in the peripheral tissues analyzed (testes and liver). An in-depth analysis of the underlying mechanisms is needed to understand the molecular basis of this phenomenon.

We performed a thorough neurodevelopmental and behavioral characterization of all generated mice, including WT males and females, hemizygous males (HEM +/Y) and homozygous (HOM +/+) and heterozygous females (HEM +/X). Our data showed that the colonies are characterized by a higher spontaneous mortality in mutated than WT mice, with a higher incidence reported in males, findings in line with the clinical profile observed in human patients. CDD is an X-linked disease with a 4:1 female:male ratio of affected patients. This sex-related distribution suggests an early lethality in males, probably during fetal life, reflected also in a much more severe course of the disease in males, often resulting in death during infancy or early adulthood [14].

The most distinctive feature of CDD, early-onset epilepsy with intractable seizures, was not recapitulated in newborn or young adult Cdkl5-mutated mice as observed in normal cage conditions up to the age of six months. No seizure-like behavior has been observed in the mice models generated to date, nor in rest condition, neither following handling or cage-changing. However, a longitudinal 24/7 monitoring in cage using more sensitive methods is necessary to exclude ictal behaviors. To the best of our knowledge, spasm-like events recorded by multichannel video-EEG have been only described in a proportion of aged (320–429 days old) heterozygous Cdkl5^R59X/+^ and Cdkl5-^KO/+^ females, with a very high heterogenicity in spasm burden [47]. Recent evidence has also demonstrated that mice lacking *Cdkl5* exhibit spontaneous epileptic EEG discharges, accompanied by increased burst activities and ictal behaviors, at postnatal day 12 only [48]. Interestingly, a wide variability in epileptic activity has been observed also among Rett syndrome mouse models, with occurrence of spontaneous seizures and type of seizures being highly dependent on the pathogenic variant of interest and the genetic background [49]. The discrepancy between humans and mice is currently difficult to explain, however the common features of CDD and RTT suggest that the epilepsy in both conditions may be due to alterations in pathways that can be affected differently to the primary gene defect (*CDKL5* or *MECP2* mutation) in humans and in mice.

We observed comparable cognitive deficits and motor impairment in male and female Cdkl5^E364X^ mice compared to sex-matched WT animals. In line with previously published findings, heterozygous females were less significantly impacted by the pathogenic variant than homozygous females and hemizygous males, possibly due to the residual activity of the wild type allele preserved in heterozygosity. From a molecular point of view in fact, male CDD patients do not have a functional Cdkl5 protein, while females, in whom the pathogenic variant always occurs in heterozygosity, have about 50 % of cells expressing the wild type protein and the remaining cells expressing the mutated allele, due to random X inactivation during development [22]. As expected, the limited studies analyzing both male and female CDKL5 mutated mice show comparable results in Cdkl5 +/+ homozygous females and Cdkl5 +/Y hemizygous male mice, with both groups of animals lacking any functional Cdkl5 protein. On the other hand, Cdkl5 X/+ heterozygous females showed an intermediate and less severe phenotype [16,21].

Behavioral analysis of Cdkl5^E364X^ mice substantially confirmed impaired motor function and altered learning and memory abilities compared to their WT counterparts, as observed in Cdkl5-KO mice [[16], [17], [18], [19], [20],^35^,^39^].

Two remarkable characteristics of the Cdkl5^E364X^ mouse are vestibular imbalance during development, as assessed by the cliff aversion test [26], and severe impairment in the rotarod test in adulthood, used to evaluate motor coordination and especially sensitive at detecting cerebellar dysfunction [50]. The cerebellum has historically been a less popular area of research compared to other brain areas in CDD animal models, despite being severely altered in patients as indicated by imaging [51], and has been the subject of very few post-mortem microscopic examination reports [52].

We therefore included the cerebellum for brain tissue analysis. Cell populations analysis of Cdkl5^E364X^ adult mice, as evaluated by the expression of cell-specific markers, confirmed the cerebellum as a severely affected area in this mouse also, reflecting our previous findings in a Cdkl5-KO mouse [31]. This is not surprising, since more than half of all CNS neurons are located in the cerebellum, and Cdkl5 is expressed in almost all neurons in the brain [42]. We found an increased expression of neurons, OPCs and mature oligodendrocyte-associated markers, possibly reflecting the developmental role of CDKL5 in neuronal migration, axon outgrowth, dendritic morphogenesis and synapse development [8]. CDKL5 regulates cell proliferation and has been indicated as a negative regulator of cerebellar granular cell precursors [53], a role which may be reflected in the increased expression of the neuron-associated marker Eno2.

CDKL5 is also involved in maintaining synaptic structure and function in the adult brain, as confirmed in Cdkl5-KO mice where synapse organization, dendritic spine stability, long-term plasticity [[54], [55], [56]] and hippocampus- and cerebellum-dependent learning and memory [31] are altered. We therefore analyzed the expression level of 84 genes involved in synaptic plasticity in the hippocampus and cerebellum, following this with a confirmatory experiment using RT-qPCR. Our results showed that *Gabra5* mRNA expression is reduced and the same trend is followed also by the expression of *Gabra1* in the cerebellum of Cdkl5 +/+ homozygous female mice, confirming the extensive involvement of this brain area in CDD. On the contrary, *Gabra1* expression is increased in male mice with the same trend observed also in *Gabra5*, a finding which may reflect the significant sex-based differences in GABAA receptor expression between males and females in humans [57], and which may also support sex-dependent disease susceptibility and progression. Sex differences in basal cerebellar synaptic physiology have been described in weanling mice, and the response to an autism-linked mutation in Gabrb3 differs between the sexes [58]. While the origin of these differences has yet to be fully understood, it has been suggested that sexually dimorphic molecular and cell biological attributes imprint cerebellar neurons in mice as early as the prepuberal stage [59].

It is well recognized that cerebellar connectivity includes cortical and limbic areas [60,61], and cerebellar molecular plasticity is essential in shaping hippocampal spatial representation [62], which is severely altered in Cdkl5-KO and Cdkl5^E364X^ mice. While not traditionally associated with epilepsy, anatomical, clinical, and electrophysiological studies point to a role of the cerebellum in seizure networks [61]. Stimulation of cerebellar areas, for example, inhibits hippocampal seizures induced by kainic acid in mice [63], thus pointing to the cerebellum as an interesting therapeutic target for seizure control [63].

*Gabra1* and *Gabra5* are inhibitory ligand-gated ion channels, thus extensively involved in inhibitory GABAergic transmission. The importance of GABA pathways regulation in CDD has been recently highlighted with the approval, in 2023, of the first adjunctive therapy for CDD to reduce the number and the severity of seizures in young patients (https://www.ema.europa.eu/en/medicines/human/EPAR/ztalmy). The active substance of this therapy is ganaxolone, a neuroactive steroid that acts through positive allosteric modulation of GABA A receptors to enhance GABA-related inhibitory tone. We previously described a GABA-glutamate molecular imbalance toward GABA hypofunction in the cerebellum of Cdkl5-KO mice [31], and we have a clear molecular hint pointing towards an impairment of GABA pathway also in the new mouse model presented in this manuscript. The importance of this balance for symptom generation is confirmed by results obtained in the p.R59X mice, in which a low-dose inhibition of glutamatergic signaling ameliorates autistic-like behaviors in these mice [20]. Finally, mutations in both *Gabra1* and *Gabra5* genes, which are dysregulated in our mice, leading to functional hypoactivity of the GABAergic system, are associated with the genetic etiology of both benign and severe epilepsy syndrome and early-onset epileptic encephalopathies [64,65].

## 5 Conclusion

The Cdkl5^E364X^ mouse, generated as tool for testing therapies for CDD patients, presents robust neurological and neurobehavioral alterations, findings which are also important in terms of primary endpoint selection for gene therapy efficacy studies, and a molecular profile related to synaptic function suggestive of a cerebellar GABAergic hypofunction. The molecular phenotype of female Cdkl5^E364X^ mice also suggests that two novel druggable targets, *Gabra1* and *Gabra5*, warrant further exploration as potential anti-epileptic therapies for CDD in future studies.

## CRediT authorship contribution statement

**C. Quadalti:** Writing – original draft, Formal analysis. **M. Sannia:** Investigation. **N.E. Humphreys:** Methodology. **V.A. Baldassarro:** Investigation, Formal analysis. **A. Gurgone:** Investigation. **M. Ascolani:** Investigation. **L. Zanella:** Investigation. **L. Giardino:** Funding acquisition, Conceptualization. **C.T. Gross:** Methodology. **S. Croci:** Investigation. **I. Meloni:** Supervision, Conceptualization. **M. Giustetto:** Writing – review & editing, Conceptualization. **A. Renieri:** Conceptualization. **L. Lorenzini:** Investigation, Conceptualization. **L. Calzà:** Writing – review & editing, Writing – original draft, Supervision, Project administration, Funding acquisition, Conceptualization.

## Ethics statement

All animal protocols described herein were carried out according to European Community Council Directive 2010/63/EU and Italian legislation (Legislative Decree 26/2014) and in compliance with the ARRIVE (Animal Research Reporting of In Vivo Experiments) guidelines and the NIH Guide for the Care and Use of Laboratory Animals. The project has been reviewed by the Animal Welfare Body of the IRET Foundation and approved by the Italian Ministry of Health (authorization no. 7/2020-PR of January 03, 2020).

## Availability of data and materials

All data generated or analyzed during this study are available in the Institutional Repository for research data (AMS Acta, University of Bologna) at the following link: https://doi.org/10.6092/unibo/amsacta/7891.

## Funding

The study was supported by the CDKL5 volunteer association (*CDKL5 Associazione di Volontariato*), Castel San Pietro Terme, Bologna, Italy.

Partially supported by the National Recovery and Resilience Plan (NRRP), project code M4C2-Investimento 1.4 CN00000041 -mRNA, in collaboration with the National Center for Gene Therapy and Drugs based on RNA Technology - financed by the 10.13039/501100000780European Union recovery package #NEXTGENERATIONEU (NGEU).

Partially supported by the 10.13039/501100003407Italian Ministry of Education, University and Research (10.13039/501100003407MIUR) funding program for Research Projects of National Relevance (PRIN), project code PRIN-10.13039/501100003407MIUR 20227HAPB7 to MG.

Partially supported by National Center for gene therapy and drugs based on RNA technology (MNRA) PNRR, Spoke 1 – Next Generation EU, Mission 4, Component 2 (M4C2), Inv. 1.4, to AR and IM; by PNC-E.3 INNOVA “Ecosistema innovativo della Salute - Missione 6 - Componente 2 - Innovazione, ricerca e digitalizzazione del servizio sanitario nazionale” - HUB 10.13039/100013288LIFE SCIENCE – Advanced Diagnostic to AR; by GENERA FSC 2014–2020 - Genoma mEdiciNa pERsonalizzatA, - POS-10.13039/501100003196Ministero della Salute T3-AN-04 to AR; to INTERVENE EU H2020-SC1-FA-DTS-2018–2020, International consortium for integrative genomics prediction - Grant Agreement No. 101016775 to AR; by SCREEN4CARE – Newborn genetic screening for prevention and treatment of rare genetic diseases,

Proposal number 101034427-1, UE, to AR.

Partially supported by PNRR THE - Tuscany 10.13039/100018696Health Ecosystem, Spoke 7, “Translational Medicine for rare, oncological and infectious diseases” financed by the 10.13039/501100000780European Union - Next Generation EU, Missione 4, Component 2, Inv. 1.5 CUP B63C22000680007 to AR and IM.

Partially supported by #NEXTGENERATIONEU (NGEU) of the Italian Ministry of Education, University and Research (MIUR), the National Recovery and Resilience Plan (NRRP), project MNESYS (PE0000006) – “A multiscale integrated approach to the study of the nervous system in health and disease” (DN. 1553 11.10.2022) to LC and LG.

The funding sources had no involvement in neither of the following: study design, conduction of the research activities, interpretation of data, preparation of the manuscript, decision to submit the article for publication.

## Declaration of competing interest

The authors declare that they have no known competing financial interests or personal relationships that could have appeared to influence the work reported in this paper.

## References

[1] Olson H.E. (2019). Cyclin-dependent kinase-like 5 deficiency disorder: clinical review. Pediatr. Neurol..

[2] Scala E. (2005). CDKL5/STK9 is mutated in Rett syndrome variant with infantile spasms. J. Med. Genet..

[3] Leonard H. (2022). CDKL5 deficiency disorder: clinical features, diagnosis, and management. Lancet Neurol..

[4] Artuso R. (2010). Early-onset seizure variant of Rett syndrome: definition of the clinical diagnostic criteria. Brain Dev..

[5] Devinsky O. (2018). Open-label use of highly purified CBD (Epidiolex®) in patients with CDKL5 deficiency disorder and Aicardi, Dup15q, and Doose syndromes. Epilepsy Behav..

[6] Kalscheuer V.M. (2003). Disruption of the serine/threonine kinase 9 gene causes severe X-linked infantile spasms and mental retardation. Am. J. Hum. Genet..

[7] Van Bergen N.J. (2022). CDKL5 deficiency disorder: molecular insights and mechanisms of pathogenicity to fast-track therapeutic development. Biochem. Soc. Trans..

[8] Zhu Y.C., Xiong Z.Q. (2019). Molecular and synaptic bases of CDKL5 disorder. Dev Neurobiol.

[9] Terzic B. (2021). Temporal manipulation of Cdkl5 reveals essential postdevelopmental functions and reversible CDKL5 deficiency disorder-related deficits. J. Clin. Invest..

[10] Kind P.C., Bird A. (2021). CDKL5 deficiency disorder: a pathophysiology of neural maintenance. J. Clin. Invest..

[11] Gao Y. (2020). Gene replacement ameliorates deficits in mouse and human models of cyclin-dependent kinase-like 5 disorder. Brain.

[12] Medici G. (2022). Expression of a secretable, cell-penetrating CDKL5 protein enhances the efficacy of gene therapy for CDKL5 deficiency disorder. Neurotherapeutics.

[13] MacKay C.I. (2021). Exploring genotype-phenotype relationships in the CDKL5 deficiency disorder using an international dataset. Clin. Genet..

[14] Jakimiec M., Paprocka J., Śmigiel R. (2020). CDKL5 deficiency disorder-A complex epileptic encephalopathy. Brain Sci..

[15] Tang S. (2017). Loss of CDKL5 in glutamatergic neurons disrupts hippocampal microcircuitry and leads to memory impairment in mice. J. Neurosci..

[16] Amendola E. (2014). Mapping pathological phenotypes in a mouse model of CDKL5 disorder. PLoS One.

[17] Wang I.T. (2012). Loss of CDKL5 disrupts kinome profile and event-related potentials leading to autistic-like phenotypes in mice. Proc Natl Acad Sci U S A.

[18] Okuda K. (2017). CDKL5 controls postsynaptic localization of GluN2B-containing NMDA receptors in the hippocampus and regulates seizure susceptibility. Neurobiol. Dis..

[19] Yennawar M., White R.S., Jensen F.E. (2019). AMPA receptor dysregulation and therapeutic interventions in a mouse model of CDKL5 deficiency disorder. J. Neurosci..

[20] Tang S. (2019). Altered NMDAR signaling underlies autistic-like features in mouse models of CDKL5 deficiency disorder. Nat. Commun..

[21] Adhikari A. (2022). Touchscreen cognitive deficits, hyperexcitability and hyperactivity in males and females using two models of Cdkl5 deficiency. Hum. Mol. Genet..

[22] Rodak M. (2022). CDKL5 deficiency disorder (CDD)-Rare presentation in male. Children.

[23] Pini G. (2012). Variant of Rett syndrome and CDKL5 gene: clinical and autonomic description of 10 cases. Neuropediatrics.

[24] Quadros R.M. (2017). Easi-CRISPR: a robust method for one-step generation of mice carrying conditional and insertion alleles using long ssDNA donors and CRISPR ribonucleoproteins. Genome Biol..

[25] Liu Y. (2019). Generation of conditional knockout mice by sequential insertion of two loxP sites in cis using CRISPR/Cas9 and single-stranded DNA oligonucleotides. Methods Mol. Biol..

[26] Feather-Schussler D.N., Ferguson T.S. (2016). A battery of motor tests in a neonatal mouse model of cerebral palsy. J. Vis. Exp..

[27] You R., Liu Y., Chang R.C. (2019). A behavioral test battery for the repeated assessment of motor skills, mood, and cognition in mice. J. Vis. Exp..

[28] Lalonde R., Strazielle C. (2011). Brain regions and genes affecting limb-clasping responses. Brain Res. Rev..

[29] de Haas R., Russel F.G., Smeitink J.A. (2016). Gait analysis in a mouse model resembling Leigh disease. Behav. Brain Res..

[30] Miedel C.J. (2017). Assessment of spontaneous alternation, novel object recognition and limb clasping in transgenic mouse models of amyloid-β and tau neuropathology. J. Vis. Exp..

[31] Sivilia S. (2016). CDKL5 knockout leads to altered inhibitory transmission in the cerebellum of adult mice. Genes Brain Behav.

[32] Borjini N. (2019). Potential biomarkers for neuroinflammation and neurodegeneration at short and long term after neonatal hypoxic-ischemic insult in rat. J. Neuroinflammation.

[33] Lorenzini L. (2016). REAC technology modifies pathological neuroinflammation and motor behaviour in an Alzheimer's disease mouse model. Sci. Rep..

[34] Prieur E.A.K., Jadavji N.M. (2019). Assessing spatial working memory using the spontaneous alternation Y-maze test in aged male mice. Bio Protoc.

[35] Gurgone A. (2023). mGluR 5 PAMs rescue cortical and behavioural defects in a mouse model of CDKL5 deficiency disorder. Neuropsychopharmacology.

[36] Ciccarelli A. (2013). Morphine withdrawal produces ERK-dependent and ERK-independent epigenetic marks in neurons of the nucleus accumbens and lateral septum. Neuropharmacology.

[37] Morello N. (2018). Loss of Mecp2 causes atypical synaptic and molecular plasticity of parvalbumin-expressing interneurons reflecting Rett syndrome-like sensorimotor defects. eNeuro.

[38] Gennaccaro L. (2021). Age-related cognitive and motor decline in a mouse model of CDKL5 deficiency disorder is associated with increased neuronal senescence and death. Aging Dis.

[39] Okuda K. (2018). Comprehensive behavioral analysis of the Cdkl5 knockout mice revealed significant enhancement in anxiety- and fear-related behaviors and impairment in both acquisition and long-term retention of spatial reference memory. PLoS One.

[40] Hector R.D. (2016). Characterisation of CDKL5 transcript isoforms in human and mouse. PLoS One.

[41] Williamson S.L. (2012). A novel transcript of cyclin-dependent kinase-like 5 (CDKL5) has an alternative C-terminus and is the predominant transcript in brain. Hum. Genet..

[42] Rusconi L. (2008). CDKL5 expression is modulated during neuronal development and its subcellular distribution is tightly regulated by the C-terminal tail. J. Biol. Chem..

[43] Fuchs C. (2015). Inhibition of GSK3β rescues hippocampal development and learning in a mouse model of CDKL5 disorder. Neurobiol. Dis..

[44] Doyle A. (2012). The construction of transgenic and gene knockout/knockin mouse models of human disease. Transgenic Res..

[45] Fazzari M. (2019). Aminoglycoside drugs induce efficient read-through of CDKL5 nonsense mutations, slightly restoring its kinase activity. RNA Biol..

[46] Javaid N., Choi S. (2021). CRISPR/Cas system and factors affecting its precision and efficiency. Front. Cell Dev. Biol..

[47] Mulcahey P.J. (2020). Aged heterozygous Cdkl5 mutant mice exhibit spontaneous epileptic spasms. Exp. Neurol..

[48] Liao W., Lee K.Z. (2023). CDKL5-mediated developmental tuning of neuronal excitability and concomitant regulation of transcriptome. Hum. Mol. Genet..

[49] Fallah M.S., Eubanks J.H. (2020). Seizures in mouse models of rare neurodevelopmental disorders. Neuroscience.

[50] Shiotsuki H. (2010). A rotarod test for evaluation of motor skill learning. J. Neurosci. Methods.

[51] Specchio N. (2023). CDKL5 deficiency disorder: progressive brain atrophy may be part of the syndrome. Cereb Cortex.

[52] Paine S.M. (2012). The neuropathological consequences of CDKL5 mutation. Neuropathol. Appl. Neurobiol..

[53] Valli E. (2012). CDKL5, a novel MYCN-repressed gene, blocks cell cycle and promotes differentiation of neuronal cells. Biochim. Biophys. Acta.

[54] Della Sala G. (2016). Dendritic spine instability in a mouse model of CDKL5 disorder is rescued by insulin-like growth factor 1. Biol Psychiatry.

[55] Pizzo R. (2016). Lack of Cdkl5 disrupts the organization of excitatory and inhibitory synapses and parvalbumin interneurons in the primary visual cortex. Front. Cell. Neurosci..

[56] Pizzo R. (2020). Structural bases of atypical whisker responses in a mouse model of CDKL5 deficiency disorder. Neuroscience.

[57] Ethiraj J. (2021). The effect of age and sex on the expression of GABA signaling components in the human hippocampus and entorhinal cortex. Sci. Rep..

[58] Mercer A.A. (2016). Sex differences in cerebellar synaptic transmission and sex-specific responses to autism-linked Gabrb3 mutations in mice. Elife.

[59] Dean S.L., McCarthy M.M. (2008). Steroids, sex and the cerebellar cortex: implications for human disease. Cerebellum.

[60] Buckner R.L. (2013). The cerebellum and cognitive function: 25 years of insight from anatomy and neuroimaging. Neuron.

[61] Rondi-Reig L. (2022). The cerebellum on the epilepsy frontline. Trends Neurosci..

[62] Rochefort C. (2011). Cerebellum shapes hippocampal spatial code. Science.

[63] Streng M.L., Krook-Magnuson E. (2021). The cerebellum and epilepsy. Epilepsy Behav..

[64] Johannesen K. (2016). Phenotypic spectrum of GABRA1: from generalized epilepsies to severe epileptic encephalopathies. Neurology.

[65] Hernandez C.C. (2019). Altered inhibitory synapses in de novo GABRA5 and GABRA1 mutations associated with early onset epileptic encephalopathies. Brain.

