# Supplementary material for: A new knockin mouse carrying the E364X patient mutation for CDKL5 deficiency disorder: neurological, behavioral and molecular profiling
Source: Heliyon. 2024 Nov 6;10(21):e40165. doi: 10.1016/j.heliyon.2024.e40165 (PMC11584566; doi:10.1016/j.heliyon.2024.e40165)
Supplement: Multimedia component 1 [file mmc1.pdf]

Supplementary Materials

Supplementary Table S1. Full list of all the genes included in the Synaptic Plasticity PCR array (PAMM-126Z, Qiagen)

GenBank ID	*Symbol*	*Description*
NM_007399	*Adam10*	A disintegrin and metallopeptidase domain 10
NM_009622	*Adcy1*	Adenylate cyclase 1
NM_009623	*Adcy8*	Adenylate cyclase 8
NM_009652	*Akt1*	Thymoma viral proto-oncogene 1
NM_018790	*Arc*	Activity regulated cytoskeletal-associated protein
NM_007540	*Bdnf*	Brain derived neurotrophic factor
NM_177407	*Camk2a*	Calcium/calmodulin-dependent protein kinase II alpha
NM_178597	*Camk2g*	Calcium/calmodulin-dependent protein kinase II gamma
NM_007664	*Cdh2*	Cadherin 2
NM_009883	*Cebpb*	CCAAT/enhancer binding protein (C/EBP), beta
NM_007679	*Cebpd*	CCAAT/enhancer binding protein (C/EBP), delta
NM_007726	*Cnr1*	Cannabinoid receptor 1 (brain)
NM_133828	*Creb1*	CAMP responsive element binding protein 1
NM_013498	*Crem*	CAMP responsive element modulator
NM_007864	*Dlg4*	Discs, large homolog 4 (Drosophila)
NM_007913	*Egr1*	Early growth response 1
NM_010118	*Egr2*	Early growth response 2
NM_018781	*Egr3*	Early growth response 3
NM_020596	*Egr4*	Early growth response 4
NM_010142	*Ephb2*	Eph receptor B2
NM_010234	*Fos*	FBJ osteosarcoma oncogene
NM_176942	*Gabra5*	Gamma-aminobutyric acid (GABA) A receptor, subunit alpha 5
NM_010305	*Gnai1*	Guanine nucleotide binding protein (G protein), alpha inhibiting 1
NM_008165	*Gria1*	Glutamate receptor, ionotropic, AMPA1 (alpha 1)
NM_013540	*Gria2*	Glutamate receptor, ionotropic, AMPA1 (alpha 2)
NM_016886	*Gria3*	Glutamate receptor, ionotropic, AMPA1 (alpha 3)
NM_019691	*Gria4*	Glutamate receptor, ionotropic, AMPA1 (alpha 4)
NM_008169	*Grin1*	Glutamate receptor, ionotropic, NMDA1 (zeta 1)
NM_008170	*Grin2a*	Glutamate receptor, ionotropic, NMDA2A (epsilon 1)
NM_008171	*Grin2b*	Glutamate receptor, ionotropic, NMDA2B (epsilon 2)
NM_010350	*Grin2c*	Glutamate receptor, ionotropic, NMDA2C (epsilon 3)
NM_008172	*Grin2d*	Glutamate receptor, ionotropic, NMDA2D (epsilon 4)
NM_133442	*Grip1*	Glutamate receptor interacting protein 1
NM_016976	*Grm1*	Glutamate receptor, metabotropic 1
NM_001160353	*Grm2*	Glutamate receptor, metabotropic 2
NM_181850	*Grm3*	Glutamate receptor, metabotropic 3
NM_001013385	*Grm4*	Glutamate receptor, metabotropic 4
NM_001081414	*Grm5*	Glutamate receptor, metabotropic 5
NM_177328	*Grm7*	Glutamate receptor, metabotropic 7
NM_008174	*Grm8*	Glutamate receptor, metabotropic 8
NM_152134	*Homer1*	Homer homolog 1 (Drosophila)
NM_010512	*Igf1*	Insulin-like growth factor 1
NM_008380	*Inhba*	Inhibin beta-A
NM_010591	*Jun*	Jun oncogene
NM_008416	*Junb*	Jun-B oncogene
NM_010623	*Kif17*	Kinesin family member 17
NM_013692	*Klf10*	Kruppel-like factor 10
NM_011949	*Mapk1*	Mitogen-activated protein kinase 1

NM_013599	*Mmp9*	Matrix metallopeptidase 9
NM_010875	*Ncam1*	Neural cell adhesion molecule 1
NM_008689	*Nfkb1*	Nuclear factor of kappa light polypeptide gene enhancer in B-cells 1, p105
NM_010908	*Nfkbib*	Nuclear factor of kappa light polypeptide gene enhancer in B-cells inhibitor, beta
NM_013609	*Ngf*	Nerve growth factor
NM_033217	*Ngfr*	Nerve growth factor receptor (TNFR superfamily, member 16)
NM_008712	*Nos1*	Nitric oxide synthase 1, neuronal
NM_016789	*Nptx2*	Neuronal pentraxin 2
NM_010444	*Nr4a1*	Nuclear receptor subfamily 4, group A, member 1
NM_008742	*Ntf3*	Neurotrophin 3
NM_198190	*Ntf5*	Neurotrophin 5
NM_008745	*Ntrk2*	Neurotrophic tyrosine kinase, receptor, type 2
NM_021543	*Pcdh8*	Protocadherin 8
NM_008837	*Pick1*	Protein interacting with C kinase 1
NM_008842	*Pim1*	Proviral integration site 1
NM_008872	*Plat*	Plasminogen activator, tissue
NM_021280	*Plcg1*	Phospholipase C, gamma 1
NM_031868	*Ppp1ca*	Protein phosphatase 1, catalytic subunit, alpha isoform
NM_013636	*Ppp1cc*	Protein phosphatase 1, catalytic subunit, gamma isoform
NM_026731	*Ppp1r14a*	Protein phosphatase 1, regulatory (inhibitor) subunit 14A
NM_019411	*Ppp2ca*	Protein phosphatase 2 (formerly 2A), catalytic subunit, alpha isoform
NM_008913	*Ppp3ca*	Protein phosphatase 3, catalytic subunit, alpha isoform
NM_011101	*Prkca*	Protein kinase C, alpha
NM_011102	*Prkcc*	Protein kinase C, gamma
NM_011160	*Prkg1*	Protein kinase, cGMP-dependent, type I
NM_009001	*Rab3a*	RAB3A, member RAS oncogene family
NM_009045	*Rela*	V-rel reticuloendotheliosis viral oncogene homolog A (avian)
NM_011261	*Reln*	Reelin
NM_009061	*Rgs2*	Regulator of G-protein signaling 2
NM_053075	*Rheb*	Ras homolog enriched in brain
NM_019812	*Sirt1*	Sirtuin 1 (silent mating type information regulation 2, homolog) 1 (S. cerevisiae)
NM_020493	*Srf*	Serum response factor
NM_177340	*Synpo*	Synaptopodin
NM_011593	*Timp1*	Tissue inhibitor of metalloproteinase 1
NM_013693	*Tnf*	Tumor necrosis factor
NM_011739	*Ywhaq*	Tyrosine 3-monooxygenase/tryptophan 5-monooxygenase activation protein, theta polypeptide
NM_007393	*Actb*	Actin, beta
NM_009735	*B2m*	Beta-2 microglobulin
NM_008084	*Gapdh*	Glyceraldehyde-3-phosphate dehydrogenase
NM_013668	*Gusb*	Glucuronidase, beta
NM_008302	*Hsp90ab1*	Heat shock protein 90 alpha (cytosolic), class B member 1

Supplementary Table S2. Details of the generation of the murine colonies of interest.

Description	Value
Pairs (n)	70
Birth (n)	48
Birth/pair ratio (%)	68.6
Pups born (n)	327
Pups weaned (n)	259
Production efficiency index (n pups weaned/ n breeding females)	0.26
Weaned to born ratio (%)	80.1

Supplementary Table S3. Gait analysis performed at 120 days with CatWalk apparatus.

			Males	Females		
			**WT (X/Y), n=36 vs. HEM (+/Y), n=28; p**	**WT (n=15) vs HET (n=16); p**	**WT (n=15) vs HOM (n=26); p**	**Overall test, p**
**Spatial values**	**Paw area**	**Front**	0.4297 (Student t test)	0.3618	0.2780	0.2920 (Kruskal-Wallis test)
				(*post hoc* Dunn's test)		
		**Hind**	0.6838 (Student t test)	0.9191	0.8932	0.9008 (One Way ANOVA)
				(*post hoc* Holm-Sidak's test)		
**Kinetic values**	**Base of Support (BOS)**	**Front**	0.2864 (Student t test)	**0.0496**	0.4476	0.0800 (Kruskal-Wallis test)
				(*post hoc* Dunn's test)		
		**Hind**	**0.0011** (Student t test)	**0.0301**	**0.0146**	**0.0205** (One Way ANOVA)
				(*post hoc* Holm-Sidak's test		
	**Stride length**	**Front**	0.4162 (Student t test)	0.3220	0.2561	0.2538 (Kruskal-Wallis test)
				(*post hoc* Dunn's test)		
		**Hind**	0.3845 (Student t test)	**<0.0001**	**<0.0001**	0.1605 (Kruskal-Wallis test)
				(*post hoc* Dunn's test)		
	**Duty Cycle (%)**	**Front**	0.7624 (Mann Whitney test)	0.1063	0.3478	0.1234 (One Way ANOVA)
				(*post hoc* Tukey's test)		
		**Hind**	0.2864 (Student t test)	**0.0496**	0.4476	0.0800 (Kruskal-Wallis test)
				(*post hoc* Dunn's test)		
**Coordination**	**Regulatory Index (%)**		0.0735 (Mann Whitney test)	0.7073	0.9289	0.6312 (Kruskal-Wallis test)
				(*post hoc* Dunn's test)		
	**Cadence**		0.0730 (Student t test)	>0.9999	>0.9999	0.9944 (Kruskal-Wallis test)
				(*post hoc* Dunn's test)		

Supplementary figures

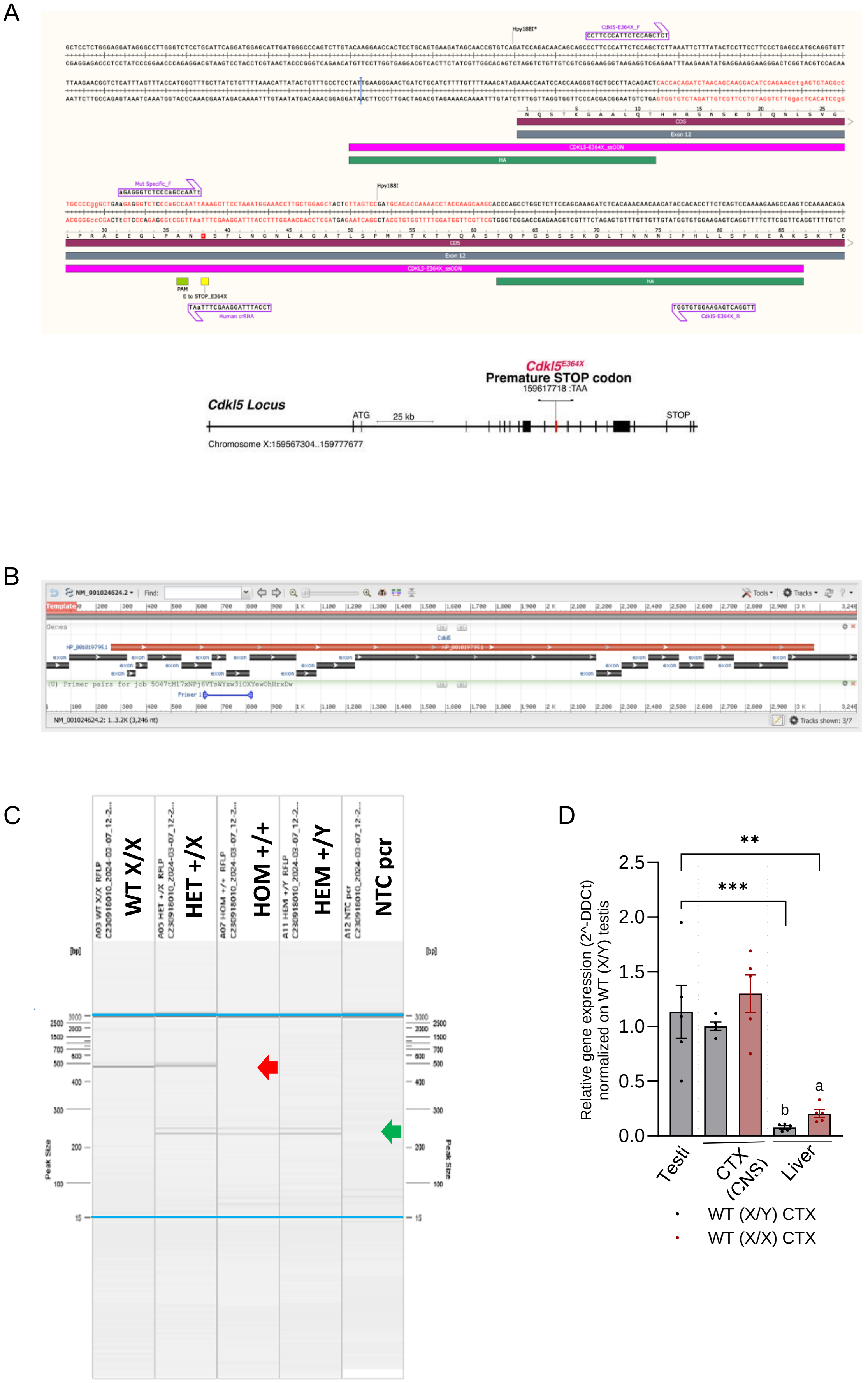

Supplementary Fig. S1. Cdkl5 mutant mice production and characterization.

(A) Strategy for the generation of the humanized Cdkl5^{E364X} mouse line. A single CRISPR crRNA was annealed with tracrRNA and combined with a homology flanked ssODN (in pink) donor coding for mutation E364X (G to T pathogenic variant) and a region of humanized sequence 46 bp downstream (the humanized sequence is depicted in red). CRISPR/Cas9 PAM in green.

In black a detail of Cdkl5 locus with respective coordinates on mouse reference genome.

(B) Position of the primers used to amplify the Cdkl5 gene independently from the presence of the humanized insert. Primers were designed using PrimerBLAST software; the output of the software is reported, with the position of the two primers sequences along the exons of the Cdkl5 gene.

(C) Representative genotyping image of gel size separation of digested (MluCI) PCR products. Gel separation analysis was performed with QIAxcel instrument and DNA High Resolution Kit (Qiagen, Hilden, Germany). Method of separation: 0M500 (injection time 10 sec.; separation time 500 sec.; injection voltage 5.0 kV; separation voltage 5.0 kV). Blu line = alignment marker; red arrow pointing the 532 bp band identifying the undigested pcr product; green arrow pointing at the two bands (250 bp and 282 bp) corresponding to the digested pcr product.

WT: only the undigested band; HET: one undigested band and one digested product; HOM and HEM: only digested products.

The original gel image of the analysis report produced by QIAxcel instrument is showed, only red and green arrows have been added in order to make results more easily readable.

(D) Relative gene expression of Cdkl5 gene in different tissues (testis, CNS(CTX), and liver) in WT male (X/Y) and female (X/X) mice normalized on the WT (X/Y) testis group. Five animals per group have been included in the qPCR analysis. One-way ANOVA followed by Tukey's *post-hoc* test was performed to compare the Cdkl5 gene expression among different tissues, while Student's t-test was used to investigate the gender effect in the same tissue. Asterisks represent the results of ANOVA with corrected multiple comparisons (*post hoc* Dunnett's test) highlighting differences between the indicated groups and the testis WT (X/Y) group (WT (X/Y) testis vs. WT (X/Y) liver, p=0.0004; WT (X/Y) testis vs. WT (X/X) liver, p=0.0016), while letters represent differences in the same tissue (a vs b, p=0.0111).

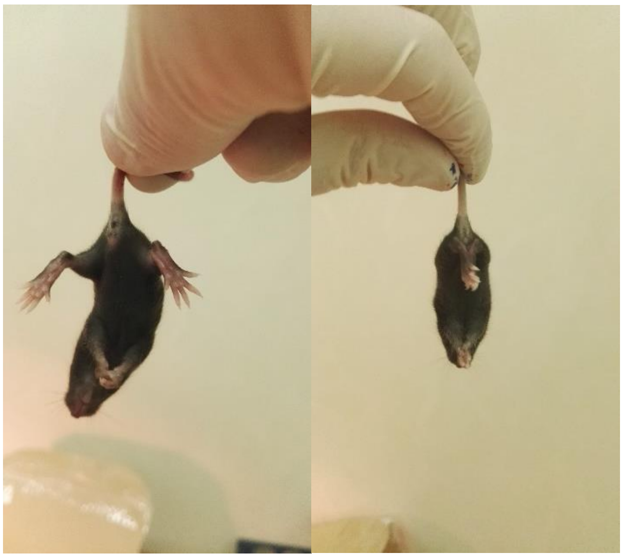

Supplementary Fig. S2 Evaluation of the clasping behaviour in mice pups. Representative images of our mice with score 0 (no clasping, on the left) and score 4 (maximum clasping score, on the right). The clasping analysis was performed in mice pups before weaning, before the genotyping results were available, therefore Scientists performing the analysis were blind to their genotype.

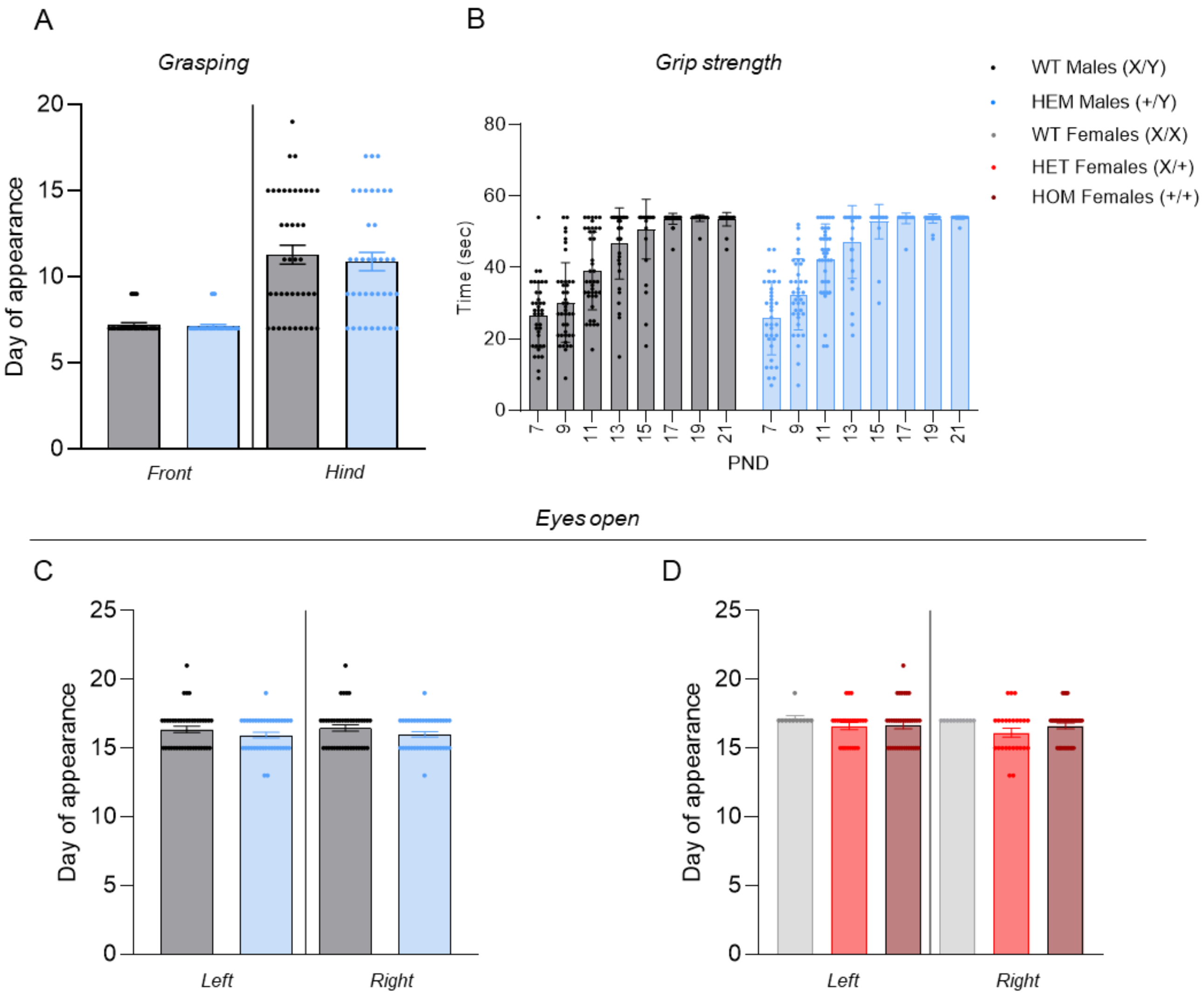

Supplementary Fig. S3. Additional data on neurological maturation assessment of Cdkl5^{E364X} mice compared to sex-matched controls.

A) Grasping analysis performed in front and hind paws of Cdkl5^{E364X} males (n=38) showing no significant difference with sex-matched WT (n=41) (Mann-Whitney test). B) Grip strength test performed on Cdkl5^{E364X} males (n=38) and sex-matched WT (n=41) from PND 7 to PND 21, showing no differences for the genotype factor (mixed-effect analysis with *post hoc* Sidak's test). C, D) Eyes open test in males (HET (+/Y) n=38, WT (X/Y) n=41) (C) and females (HOM (+/+) n=36, HET (X/+) n=25, WT (X/X) n=11) (D), showing no differences in the day of appearance when compared to sex-matched controls.

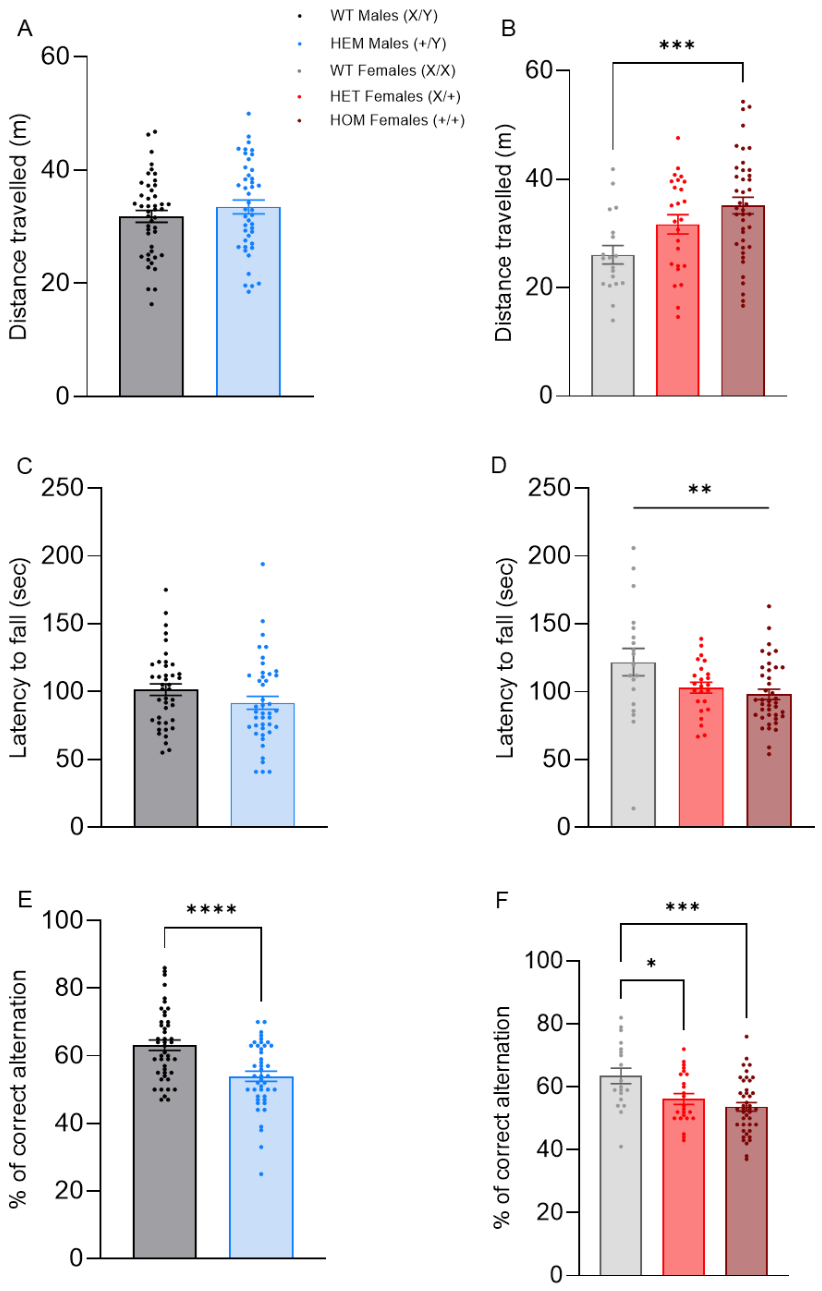

Supplementary Fig. S4. Locomotion, rotarod and Y-maze test at 80 days of age.

(A, B) Spontaneous locomotion in an open-field arena in all genotypes in male (A) and female (B) mice, expressed as distance traveled. Statistical analysis showed no significance between male genotypes (HEM (+/Y), n=44; WT (X/Y), n=45), while female (one-way ANOVA, p=0.0021, F=6.661) HOM (+/+) (n=41) showed a significantly higher activity than sex-matched WT (n=19) (*post hoc* Tukey's test, p=0.0014); no difference was observed between HET (X/+) (n=25) vs WT or HOM. Asterisks in females represent the results of Tukey's test.

(C, D) Sensorimotor coordination in the rotarod test in all genotypes in male (C) and female (D) mice, expressed as latency to fall. Males showed no significant difference (Mann-Whitney test on WT (X/Y) (n=41) vs. HEM (+/Y) (n=43)); females (one-way ANOVA, p=0.0144, F=4.471) showed a significant impairment in the comparison between HOM (+/+) (n=41) vs WT (X/X) (n=19) (*post hoc* Tukey's, p=0.0110), while no difference was observed in HET (X/+) (n=24). Asterisks represent the results of multiple comparisons one-way ANOVA with Tukey's test.

(E, F) Spatial memory evaluated by Y-maze test in male (E) and female (F) mice, expressed as % of correct alternations. Hemizygous males (n=44) showed a clear impairment when compared to WT (X/Y) (n=45) (Student's t-test, $p<0.0001$). Also, Cdkl5 females showed a significant impairment (one-way ANOVA, $p=0.0008$, $F=7.736$; *post hoc* Tukey's test, HET (X/+) (n=24) vs WT (X/X) (n=19), $p=0.0267$; HOM (+/+) (n=42) vs WT (X/X), $p=0.0005$). Asterisks represent the results of multiple comparisons one-way ANOVA with *post hoc* Tukey's test.
*p<0.05; **p <0.005; ***p <0.001; ****p<0.0001.
All data are expressed as mean ± SEM.

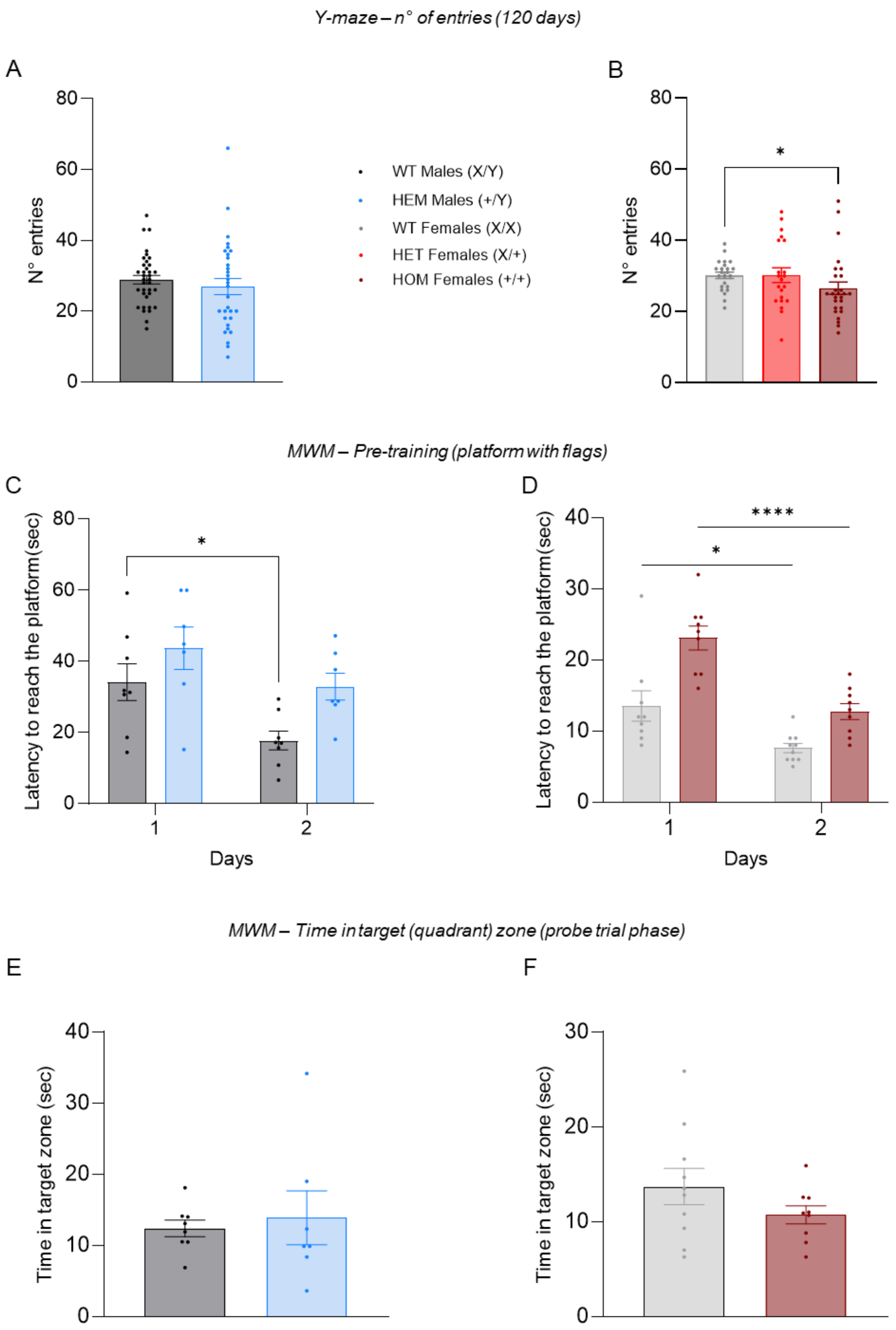

Supplementary Figure S5. Additional data for Y-maze and Morris water maze tests.

The number of entries registered during the Y-maze test are shown, in male mice (A) and female (B) mice. The statistical analysis was performed with Mann-Whitney test for male comparison (not significant; WT (X/Y), n=36; HEM (+/Y), n=31), and with Kruskal-Wallis test with *post hoc* Dunn's test, with asterisk representing the corrected multiple comparison result (WT (X/X) (n=23) vs HOM (+/+) (n=27), p=0.0235). No significant comparison was observed for female HET (X/+) (n=21).

The pre-training phase of the MWM test was evaluated through the parameter “latency to reach the platform”, which in this phase is marked by flags. The statistical analysis was performed with two-way ANOVA with *post hoc* Sidak’s test for both males (C) and females (D) comparisons. The factors analyzed are genotype (males, WT (X/Y), n=8; HEM (+/Y), n=7, $p=0.0109$, $F(1, 26)=7.512$; females, WT (X/X), n=10, HOM (+/+), n=9, $p<0.0001$, $F(1, 33)=25.16$) and time (males, $p=0.0056$, $F(1, 26)=9.129$; females, $p<0.0001$, $F(1, 33)=30.75$); in both cases the interaction is not significant.
Asterisks represent the result of corrected multiple comparisons tests.
The time in target (quadrant) area is shown for males (E) and females (F). For both males (WT(X/Y), n=8, vs HEM(+/Y), n=7), and females (WT (X/X), n=10, vs HOM (+/+), n=9) comparisons, Student’s t-test was performed and no significant differences were observed.
*$p<0.05$; **$p<0.005$; ***$p<0.001$; ****$p<0.0001$.

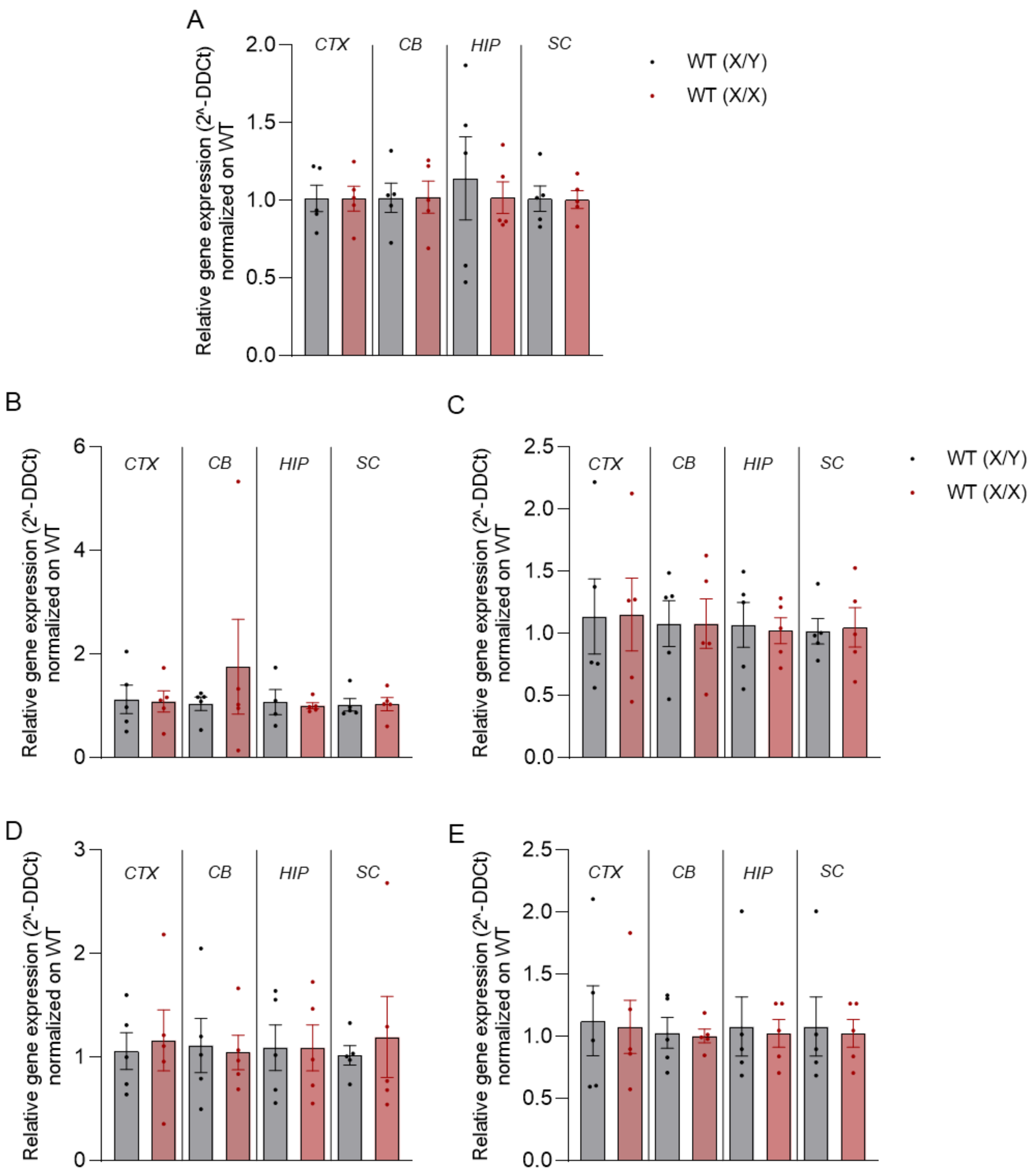

Supplementary Fig. S6. Additional statistical analysis on Cdkl5, Eno2, GFAP, Pdgfra, Mbp gene expression.

Bar graphs show the individual distribution of gene expression data in WT males and females for A) Cdkl5, B) Eno2, C) GFAP, D) Pdgfra, E) Mbp genes, respectively. Five animals were included for each group. Comparison between male and female groups for each gene of interest was performed with Student's t-test, no statistical significance was obtained.

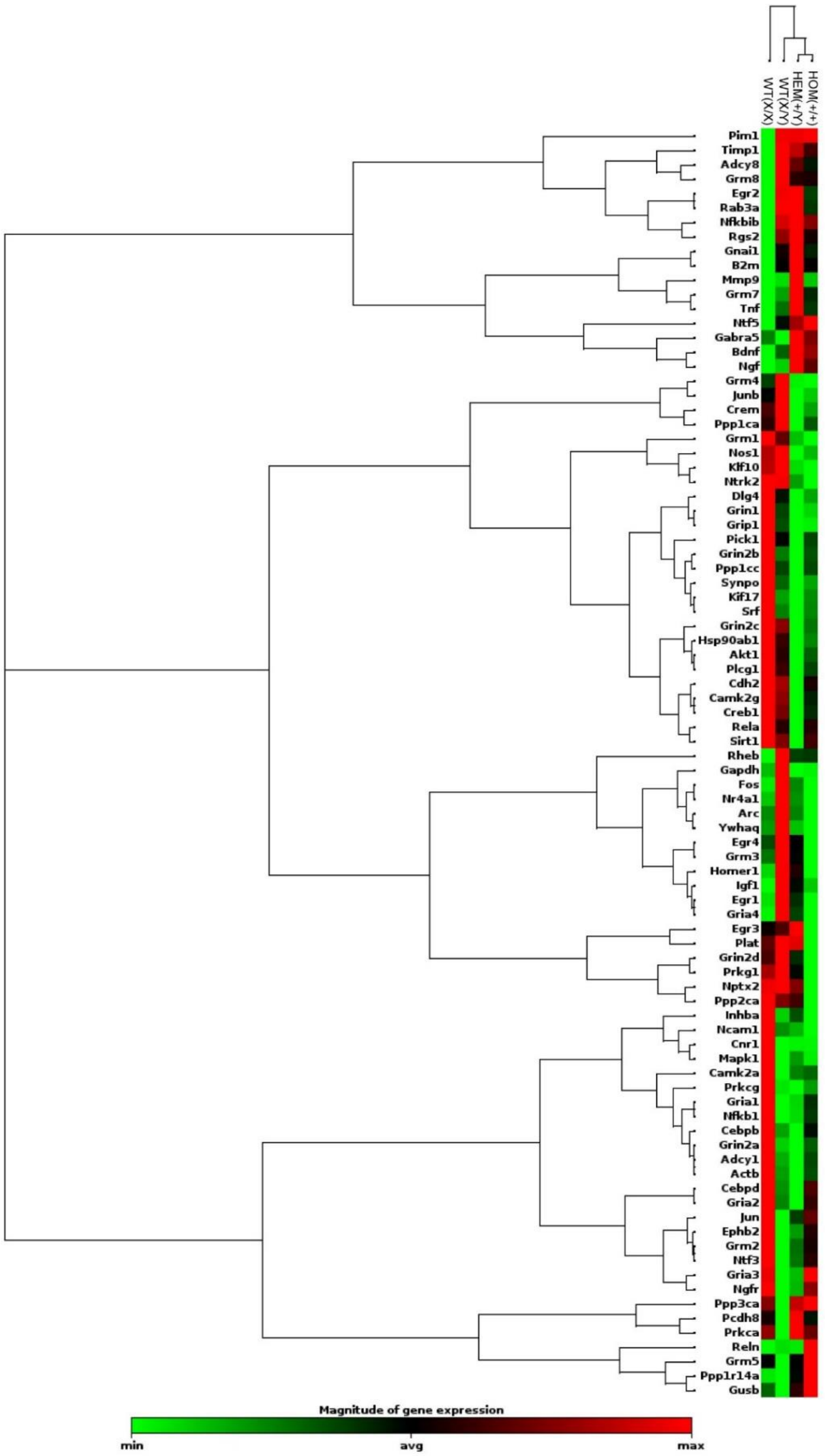

Supplementary Figure S7. PCR array clusterization analysis in hippocampus.

Graph shows the relative expression within each gene throughout the different groups, indicating the magnitude of expression in a color scale, with green representing the minimum and red the maximum of expression. Lines represent the clusterization of both experimental groups and genes. Gene symbols are included in the figure.

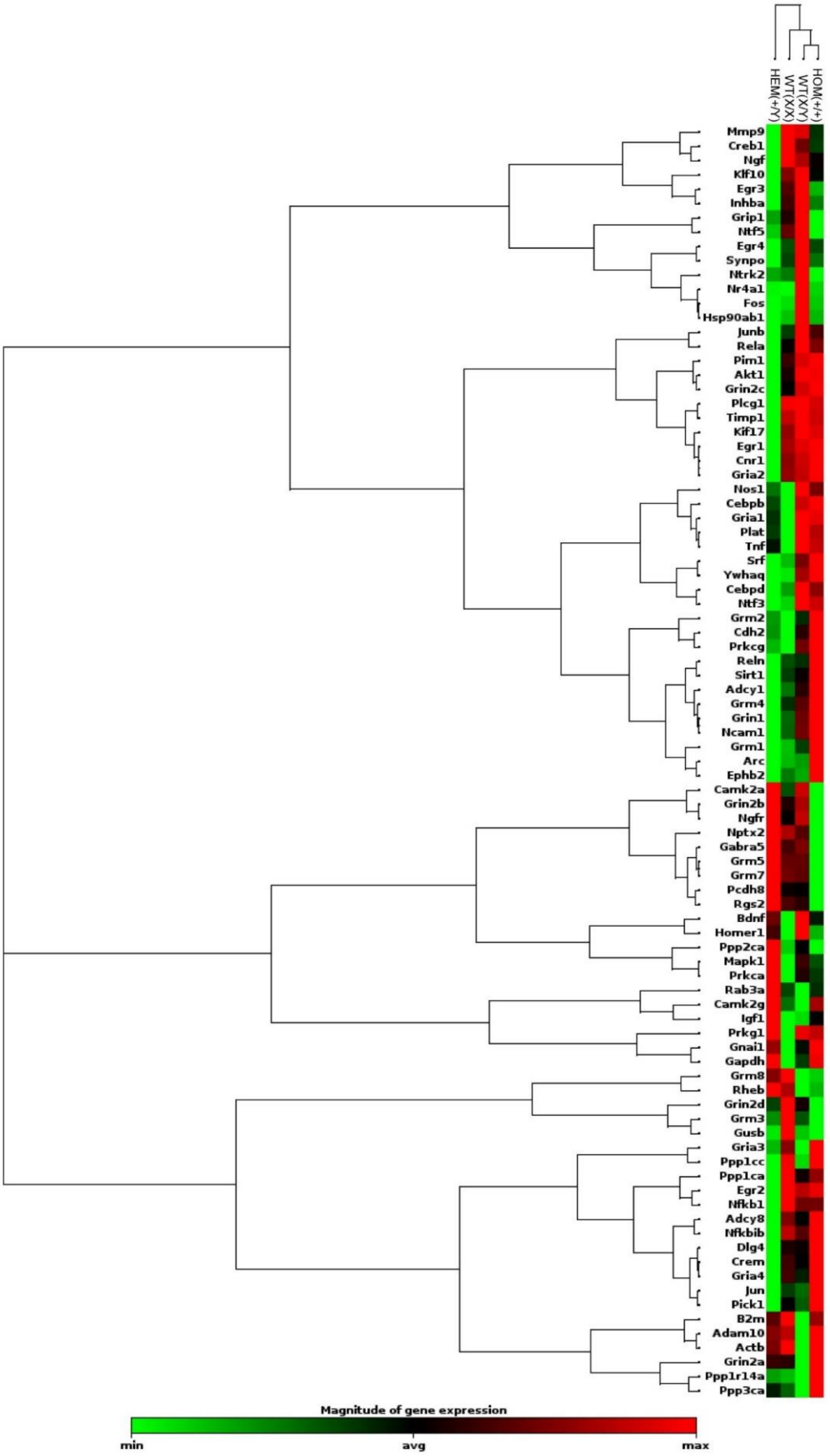

Supplementary Figure S8. PCR array clusterization analysis in cerebellum.

Graph shows the relative expression within each gene throughout the different groups, indicating the magnitude of expression in a color scale, with green representing the minimum and red the maximum of expression. Lines represent the clusterization of both experimental groups and genes. Gene symbols are included in the figure.
